# Synergistic therapy of Chinese herbal medicine and gut microbiota modulation for post-stroke cognitive recovery: focus on microbial metabolite and immunoinflammation

**DOI:** 10.3389/fmicb.2025.1623843

**Published:** 2025-08-14

**Authors:** Shihui Ge, Shuangli Zhang, Linjing She, Tianning Gu, Shicong Wang, Xin Huang, Lanlan Wang, Mingsan Miao

**Affiliations:** ^1^Department of Pharmacy, Henan University of Traditional Chinese Medicine, Zhengzhou, China; ^2^Collaborative Innovation Center of Research and Development on the Whole Industry Chain of Yu-Yao, Zhengzhou, China; ^3^Academy of Chinese Medicine, Henan University of Chinese Medicine, Zhengzhou, China; ^4^Institute of Essential Oils of Chinese Medicine, Henan Academy of Traditional Chinese Medicine, Zhengzhou, China

**Keywords:** post-stroke cognitive impairment, microbiota-gut-brain axis, immunoinflammation, Chinese medicine, probiotics

## Abstract

Post-stroke cognitive impairment (PSCI), a common complication following stroke, significantly impacts patients' quality of life and rehabilitation. Recent studies have highlighted the role of gut microbiota and their metabolites in modulating immunoinflammation and cognitive function via the gut-brain axis. Traditional Chinese medicine (TCM) and microbiota interventions including probiotics and fecal microbiota transplantation, have shown potential in reshaping gut microbial communities and metabolite profiles. Some studies suggest that combining these approaches via identical or related therapeutic mechanisms may yield enhanced efficacy in treating Post-Stroke Cognitive Impairment (PSCI). These findings establish a theoretical foundation for future research and clinical practice. This review systematically examines the mechanistic role of gut microbial metabolites in neuroimmune modulation and comprehensively evaluates the therapeutic potential of combined TCM and microbiota-targeted therapies for PSCI, adopting a multifactorial approach that addresses neuroinflammation, microbial dysbiosis, and metabolic dysregulation.

## Introduction

Post-stroke cognitive impairment (PSCI), affecting 4.4–73% of stroke survivors, poses a significant global public health challenge due to its high prevalence and debilitating consequences (Rost et al., 2022; Gallucci et al., 2024). Despite advancements in stroke management, over one-third of survivors continue to experience progressive cognitive decline, predominantly manifesting as executive dysfunction, attention deficits, and memory impairment (Aam et al., 2020). The pathogenesis of PSCI is now recognized to involve disrupted neural networks from cerebrovascular injury, β-amyloid deposition, microglial activation-driven neuroinflammation, and cholinergic system dysregulation (Wang et al., 2016; Cho et al., 2021; Park et al., 2021). Current therapeutic strategies, such as acetylcholinesterase inhibitors, N-methyl-D-aspartate receptor (NMDA) antagonists, and calcium channel blockers, demonstrate only transient symptomatic efficacy, thereby underscoring the urgent demand for novel multi-targeted interventions addressing the complex pathophysiology of PSCI (Quinn et al., 2021).

The gut microbiota has been shown to play a role in regulating the central nervous system (CNS) regulation via the gut-brain axis. Gut microbial ecosystems actively produce bioactive metabolites such as short-chain fatty acids (SCFAs) and serotonin (5-HT), which communicate with the brain through neural, endocrine, and immune pathways (Romano et al., 2015; Zheng et al., 2020). Sympathetic hyperactivity after stroke and hypothalamic-pituitary-adrenal (HPA) axis dysfunction disrupt gut barrier integrity, exacerbating dysbiosis and dysbiosis-derived metabolites, such as lipopolysaccharides (LPS) and Trimethylamine n-oxide (TMAO), in turn aggravate neuroinflammation and blood-brain barrier (BBB) leakage, thereby forming a vicious cycle (Keller et al., 2017; O'Riordan et al., 2022). These mechanisms collectively underpin the therapeutic rationale for microbiota-targeted interventions in PSCI.

Traditional Chinese Medicine (TCM) demonstrates unique advantages in PSCI treatment. Imbalanced Qi (vital energy) and blood flow, or phlegm-stasis obstruction, may impair the function of Yuan Shen (Primordial Spirit), and cause neurological damage manifesting as cognitive impairment. For instance, It was discovered that Wen Fei Jiang Zhuo formula evidently reduced vascular dementia symptoms via microbiota-gut-brain axis modulation, based on the theory of Wen Fei Jiang Zhuo (warming the lungs to dispel turbidity) (Zhan et al., 2023). Notably, medicinal herbs, such as *Pueraria lobata* (Willd.) Ohwi, *Scutellaria baicalensis* Georgi, *Lycium barbarum* L., contain microbiota-modulating fibers and neuroprotective compounds, such as puerarin, baicalin, Lycium barbarum polysaccharide, which enhance synaptic plasticity and suppress inflammation (Chen et al., 2019a; Liu et al., 2020b). Concurrently Probiotics, prebiotics, synbiotics, and postbiotics (PPSP) directly modulate gut microbiota, mitigating neuronal damage and cognitive deficits (MAO et al., 2024). Recent studies have revealed that gut microbiota metabolites, such as SCFAs and TPH (TPH) derivatives, regulate microglial polarization and synaptic plasticity via the neuro-immune-metabolic axis, mediating post-stroke cognitive recovery. Combined with microbial therapies, these natural compounds may synergistically regulate the “microbiota metabolite-neuroimmune” axis, overcoming limitations of single-target pharmacological approaches.

Therefore, we reviewed the current research advancements and proposes a hypothesis: combining TCM with microbiota-targeted interventions may regulate core inflammatory mechanisms and improve neuronal energy supply through metabolites, thereby coherently integrating localized anti-inflammatory effects with systemic metabolic repair. This article comprehensively analyzed the progress of clinical observations and animal experiments.

## Immunoinflammation and PSCI

Neuroinflammation and oxidative stress critically impair neuronal function and synaptic plasticity, underpinning spatial disorientation and memory deficits in PSCI (Zhang et al., 2021b). Activated microglia, as brain-resident immune cells, may release chemokines in response to surrounding cytokine signaling, and recruit polarized lymphocytes across the BBB, amplifying post-stroke neurotoxicity (Rutsch et al., 2020). Pathogenic damage-associated molecular patterns (DAMPs) from necrotic cells may trigger microglial and astrocytic activation, perpetuating neurotoxic cascades (Iadecola et al., 2020). A study has found that microglial pro-inflammatory mediators in ischemic stroke, such as tumor necrosis factor-alpha (TNF-α), interleukin-1 beta (IL-1β), interleukin-6 (IL-6), high mobility group box 1 (HMGB1), induce NADPH oxidase-mediated superoxide production, compromising BBB integrity and perpetuating nerve damage (Yang et al., 2022). Gut dysbiosis exacerbates further central inflammation via LPS produced by Gram-negative bacteria (Escherichia coli, Bacteroides fragilis), which penetrate the compromised BBB to activate toll-like receptor 4 (TLR4) signal (Martin et al., 2018; Xu et al., 2020a). Notably, Poly (ADP-ribose) polymerase 9 (PARP9), an ADP-ribosyl transferase regulating apoptosis and inflammation, emerges in secondary brain injury. Following cerebral cortical infarction, PARP9 expression was upregulated in the non-ischemic thalamus and hippocampus of hypertensive rats. PARP9 knockdown alleviated neuronal apoptosis and neuroinflammation via PI3K pathway activation, promoting cognitive recovery (Xu et al., 2020a; Liao et al., 2025). A systematic review and meta-analysis revealed that significant biomarkers of PSCI were identified in peripheral blood. PSCI patients exhibited markedly elevated levels of inflammatory markers (e.g., IL-6, C-reactive protein CRP), which showed a negative correlation with cognitive scores (standardized mean difference SMD = 0.46, correlation coefficient *r* = −0.25) (Tack et al., 2025). In essence, stroke is a vascular injury at its core. Chronic inflammation in microvessels and increased endothelial activation elevate BBB permeability, facilitating the infiltration of inflammatory factors such as interleukins (ILs), matrix metalloproteinases (MMPs), TNF-α, TLR4, and CRP. These processes may exacerbate white matter disruption and amplify neuroglial inflammation (Cipollini et al., 2019). Consequently, heightened intravascular inflammation and oxidative stress levels may indicate an elevated risk of developing PSCI.

## Microbial metabolites and the gut-brain axis

Recent studies have revealed that PSCI patients frequently exhibit gastrointestinal dysfunction alongside classical neurocognitive deficits. Concurrently, advancements in the gut-brain axis framework highlight multidimensional interactions involving neuroendocrine, immune, and microbial metabolic processes. This raises the critical question of how gut microbiota-derived neuroactive metabolites (e.g., SCFAs and TPH derivatives) exert regulatory effects on PSCI progression. Specifically, what roles do these metabolites play in modulating post-stroke cognitive decline through neuroimmune pathways and BBB permeability mechanisms?

### SCFAs

PSCI patients exhibit reduced gut microbiota α-diversity, Fusobacterium enrichment, and diminished SCFA production, compared to non-PSCI controls. An increase in Fusobacterium and a deficiency in microbial-derived short-chain fatty acids (SCFAs) were significantly associated with PSCI. Models based on gut microbiota and SCFA profiles could accurately predict PSCI at 3 months or beyond post-stroke early after stroke onset (Wang et al., 2022). Studies revealed that SCFAs (acetate, propionate, butyrate) from dietary fiber fermentation modulated microglial activation, neurotrophic factors, BBB integrity, and apoptosis via the immune and circulatory systems, and thus affected post-stroke cognitive impairment (Liu et al., 2020c; Agus et al., 2021; Zhou et al., 2021). Butyrate is a key neuromodulator, inhibiting microglia overactivity through Akt phosphorylation by oral sodium butyrate, reducing neuronal apoptosis and cerebral infarct size, decreasing the degree of cerebral edema, and improving cognitive performance after stroke (Liu et al., 2022a). Moreover, Dynamic post-stroke SCFA fluctuations (early acetate/propionate decline, sustained butyrate/valerate reduction, and transient isobutyrate/isovalerate increase) is identifed to be correlated with cognitive trajectories (Chen et al., 2019b). Mechanistically, acetate activate GPR41 and inhibite MAPKs phosphorylation, thereby suppressing the activation of p38, JNK, ERK, and NF-κB signaling pathways. This cascade downregulate P65 expression and reduce pro-inflammatory cytokine release (Liu et al., 2020a). Additionally, acetate preserves gut barrier integrity and inhibits IL-1β/IL-6 production, while Bacteroides abundance is inversely correlated with systemic inflammation. Notably, butyrate may promote oligodendrocyte differentiation and remyelination in multiple sclerosis models, whereas chronic IL-1β/IL-18 elevation in an ischaemic model predicte long-term cognitive deficits (Chen et al., 2019c,d).

### TMAO

TMAO is derived from gut microbiota and generated from dietary choline via hepatic flavin monooxygenase (FMO3), represents a novel predictive and therapeutic target for PSCI. A study showed after adjusting for potential confounders, multivariate logistic analysis demonstrated that elevated plasma TMAO levels independently predicted post-stroke cognitive impairment (95% CI: 1.335–8.178; *P* = 0.010) (Zhu et al., 2020; Tu and Xia, 2024). Analysis of 351 first-episode IS patients revealed that elevated plasma TMAO at admission correlated with worse neurological outcomes and higher mortality at 3 months. Each 1 μmol/L increase in TMAO raised severe neurological damage risk by 21%, demonstrating a positive association with neurological injury severity and mortality (Zhang et al., 2021a). Preclinical studies has confirmed TMAO neurotoxicity. Exogenous TMAO exacerbates astrocyte overactivation and glial scar formation, and promotes neuroinflammation in the middle cerebral artery occlusion/reperfusion (MCAO/R) model (Brunt et al., 2020; Su et al., 2021). Moreover, endoplasmic reticulum stress-induced synaptic plasticity impairment involves TMAO-mediated protein misfolding (Govindarajulu et al., 2020). Aged mice exhibited significantly higher levels of trimethylamine N-oxide (TMAO) compared to younger mice. This was accompanied by increased pro-inflammatory cytokines (e.g., IL-6, and TNF-α) and elevated astroglial activation markers. Furthermore, young mice fed a long-term high-TMAO diet performed significantly worse on cognitive tests (e.g., novel object recognition), suggesting that TMAO directly impairs cognitive function (Brunt et al., 2020). Mechanistically, TMAO acts as an upstream driver of vascular endothelial dysfunction, adversely affecting nitric oxide (NO) release and function through multiple pathways (Fu et al., 2024). TMAO induces vascular endothelial dysfunction and vascular inflammation via activation of inflammasomes, as well as the mitogen-activated protein kinase (MAPK), extracellular signal-regulated kinase (ERK), and nuclear factor kappa-B (NF-κB) signaling pathways (Sun et al., 2016b). In aged traumatic rats, TMAO reduced methionine sulfoxide reductase expression, thereby enhancing reactive oxygen species (ROS) accumulation and nuclear factor kappa-B (NF-κB)-driven neuroinflammation (Meng et al., 2019). Conversely, memantine combined with Lactobacillus plantarum lowered hippocampal β-Amyloid (Aβ) deposition and TMAO levels in Alzheimer's disease (AD) mice, preserving neuronal integrity and plasticity (Wang et al., 2020).

### Secondary bile acids

Cholic acid (CA) has been demonstrate the capacity to diffuse through phospholipid bilayers and subsequently cross the BBB. CA treatment significantly increased the phosphorylation levels of BDNF, CREB, PI3K, Akt, MAPK, and Erk in both *in vitro* neurovascular unit (NVU) models and their oxygen-glucose deprivation/reoxygenation (OGD/R) counterparts. These results indicate that CA restores BBB integrity and neuronal phenotypes in the neurovasculature by activating the BDNF-TrkB-MAPK/Erk and BDNF-TrkB-PI3K/Akt signaling pathways, which modulated neuroinflammation, oxidative injury, and growth factor regulation (Li et al., 2020). Secondary bile acids, predominantly deoxycholic acid (DCA) as the most abundant species, are derived from bacterial modification of primary bile acids such as CA and chenodeoxycholic acid in the intestinal tract. Bile acid dysregulation links gut microbiota to neuroinflammation: Cognitive impairment in Alzheimer's disease has been found to be associated with levels of primary (liver-generated) bile acids and elevated levels of secondary (microbiota-modified) bile acids (MahmoudianDehkordi et al., 2019). Given that bile acids regulate lipid metabolism, energy homeostasis, and gut barrier function, and also influence neuroinflammation, this shift in bile acid profile may contribute to disease mechanisms (Collins et al., 2023; Xing et al., 2023). In a rat model of acute stroke, the administration of tauroursodeoxycholic acid (TUDCA) 1 h post-ischemia resulted in elevated cerebral bile acid levels, improved neurological function, and ~50% reduction in infarct volume at 2 and 7 days post-reperfusion. TUDCA has been found to markedly suppress endoplasmic reticulum (ER) stress, decreas the number of TUNEL-positive brain cells and mitochondrial swelling, and partially inhibit caspase-3 processing and substrate cleavage, thereby exerting neuroprotective effects (Rodrigues et al., 2002; Chen et al., 2020). Moreover, INT-777 -mediated activation of the G protein-coupled bile acid receptor Gpbar1 (TGR5) upregulates Brca1/Sirt1 signaling pathway, which in turn attenuates BBB disruption post-MCAO (Keitel et al., 2010; Liang et al., 2020a). Hydrophilic ursodeoxycholic acid (UDCA, as a therapeutic bile acid) exerts dual neuroprotection by activating TGR5 and inhibiting NOD-like receptor family pyrin domain containing 3 (NLRP3)/IL-1β, reducing infarct size and cognitive deficits (Zhang et al., 2024). Systemic bile acid alterations have also been implicated in other neuropsychiatric disorders, including hepatic encephalopathy, AD and depression (Dantas Machado et al., 2023; Chen et al., 2024; Jia et al., 2024).

### TPH derivatives

**Figure 1:**
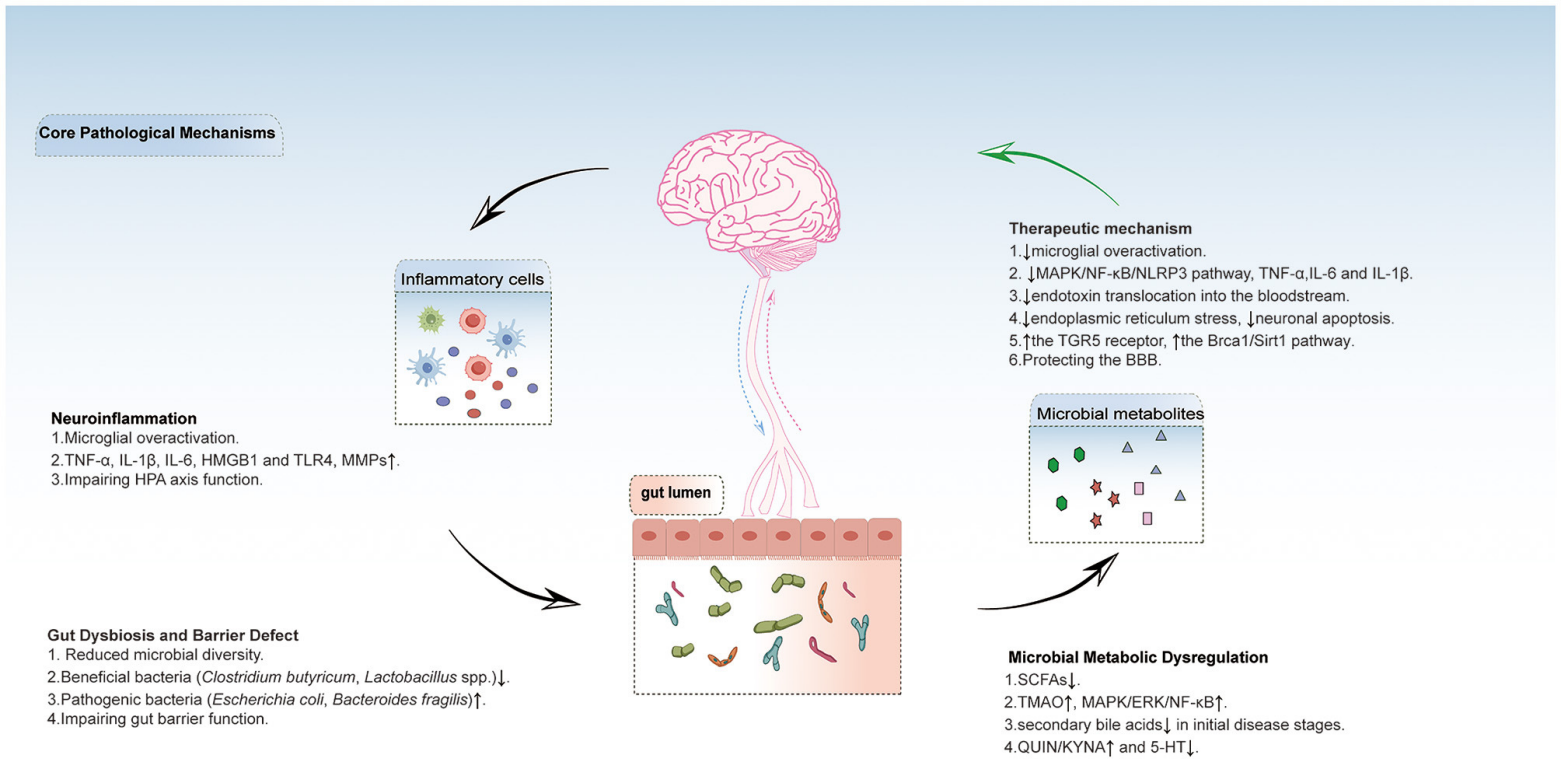
This chematic diagram shows how post-stroke cognitive impairment (PSCI) interacts with the gut microbiome through via gut-brain axis. Post-stroke neuroinflammation and gut microbiota disruption form a bidirectional vicious cycle: Neuroinflammation damages the intestinal barrier and microbiota balance, reducing SCFAs while increasing harmful metabolites (TMAO, QUIN). These metabolites enter the brain, further activating microglia and exacerbating neuroinflammation, ultimately damaging the blood-brain barrier and neurons. Supplementing beneficial metabolites can inhibit inflammatory pathways through their receptors, alleviating neuroinflammation, protecting the blood-brain barrier, and breaking this cycle. TNF-α, tumor necrosis factor-α; IL-6, interleukin-6; IL-1β, interleukin-1β; HMGB1, high mobility group box 1; MMPs, matrix metalloproteinases; HPA axis, hypothalamic-pituitary-adrenal axis. SCFAs, short chain fatty acids;TMAO, trimethylamine n-oxide; ERK, extracellular signal-regulated kinase; QUIN, quinolinic acid; KYNA, kynurenine acid; 5-HT, serotonin; MAPK, mitogen-activated protein kinase; NF-κB, nuclear factor kappa-light-chain-enhancer of activated B cells; NLRP3, NOD-like receptor family pyrin domain containing 3; TGR5, the G protein-coupled bile acid receptor, Gpbar1;BBB, blood brain barrier.

Microbial TPH metabolites, such as indoles, kynurenines, and 5-HT, may modulate neuroimmune balance. Firstly, the kynurenine pathway balance is critical for neuroinflammation. A prospective clinical study involving 23 stroke patients demonstrated significant correlations between baseline serum levels of quinolinic acid (QUIN), kynurenic acid (KYNA), the QUIN/KYNA ratio, and post-stroke cognitive performance (Cogo et al., 2021). Both QUIN and KYNA participate in modulating synaptic plasticity. Notably, gut dysbiosis may disrupt the QUIN/KYNA balance, where elevated QUIN levels induce excitotoxicity through NMDA receptor activation, whereas KYNA exerts neuroprotective effects (Hertelendy et al., 2018). Clinical studies demonstrated a negative correlation between serum 3-hydroxykynurenine (3-HK) levels and Montreal Cognitive Assessment (MoCA) scores in stroke patients (Braidy and Grant, 2017; Sandvig et al., 2024). Furthermore, AD patients exhibit TPH depletion and elevated KYNA/TPH ratios, which are associated with accelerated cognitive decline (Roth et al., 2021). Another study on ischemic stroke patients has shown that the KYNA/TPH ratio is positively correlated with stroke severity. Simultaneously, metabolomic analysis has revealed elevated serum lactate and glutamate levels, along with reduced TPH levels in these patients. Moreover, statistical correlations have indicated a robust link between elevated brain QUIN levels and the severity of autism-related behavioral deficits, as well as neurotransmitter imbalances. Oral administration of *Bifidobacterium* CCFM1077 effectively modulated QUIN concentrations in the brain, rebalanced the glutamate to γ-aminobutyric acid (GABA) ratio in the central nervous system, and simultaneously reduced cerebellar microglial activation (Kong et al., 2022). Additionally, numerous studies have found that indole-3-lactic acid can activate the aryl hydrocarbon receptor (AhR), which suppresses IL-1β, IL-6 and repairs neuroimmune homeostasis (Qian et al., 2024). Indole-3-carboxaldehyde (I3C), a gut microbiota-derived metabolite, functions as a gut-brain signaling molecule through the AhR pathway. I3C attenuates NF-κB activation and NLRP3 inflammasome formation. This suppresses neuroinflammation and promotes hippocampal neurogenesis, ultimately reducing host susceptibility to stress (Chen et al., 2025b). Moreover, Certain bacteria in the gut, such as *Escherichia coli, Lactobacillus* and *Bifidobacterium*, can participate in the metabolism of TPH, which is a precursor for serotonin (5-HT) synthesis. Research has found that Epimedium total flavonoids can improve cognitive function in the PSCI rat model, increase the levels of acetylcholine, DA, 5-HT, and norepinephrine, while decreasing the levels of amyloid beta 1-42 (Aβ1-42) and neuron-specific enolase (NSE) (Yang et al., 2024). SCFAs stimulate gut 5-HT synthesis, improving barrier function and reducing neuroinflammation.This also suggests the existence of a dynamic regulatory network among microbial metabolites (Silva et al., 2020). Figure 1 shows how post-stroke cognitive impairment (PSCI) interacts with the gut microbiome through via gut-brain axis.

## Impact of Chinese medicines and their components on gut microbial communities

An important strategy for intestinal flora regulation and barrier protection includes restoring intestinal flora homeostasis and strengthening intestinal barrier integrity to attenuate systemic inflammation and cognitive decline (Supplementary Table S1). Schisandrin (Zhang et al., 2022; Fu et al., 2023) and Panax notoginseng saponins (Hu et al., 2025) increased Firmicutes/Bacteroidetes ratios (F/B), enriched the abundance of *Eubacterium*, and enhanced intestinal barrier function while reducing the endotoxin leakage. Panax notoginseng saponins (Hu et al., 2025) elevated the abundance of *Lactobacillus reuteri*, promoted histidine synthesis and alleviated ischemic neuronal injury. Walnut-derived peptide LPLLR (Qi et al., 2023), *Pueraria lobatae* and *Ligusticum chuanxiong* (Chen et al., 2019a) upregulated tight junction proteins, such as Zona Occludens 1 (ZO-1), Claudin-1 and mucin-2 (MUC2). These findings collectively suggest that such interventions reduce gut permeability and systemic inflammation. Meanwhile, probiotics like *C. butyricum* (*C. butyricum)* and *Lactobacillus* increased the abundance of bile acids. Especially butyrate, inhibited neuroinflammation via the gut-brain axis and TLR4/MyD88/NF-κB inhibition (Song et al., 2024). Baicalein, eucommiae cortex polysaccharides (Sun et al., 2022), and Naoxintong capsules (Li et al., 2025) inhibited TLR4/NF-κB activation and reduced hippocampal TNF-α, IL-1β, and IL-6 levels, thereby attenuating microglia overactivation and neuronal apoptosis. Huanglian Jiedu Decoction inhibited Cyclooxygenase-2 (COX-2)/5-lipoxygenase (5-LOX) pathways, reducing Aβ deposition and tau hyperphosphorylation in AD models (Gu et al., 2021). Gastrodia elata Bl. elevates prefrontal 5-HT, DA, and 3,4-dihydroxyphenylacetic acid (DOPAC), improving stress-induced depression and cognitive deficits in a stress-induced model. As well it modulates neuroprotection through metabolites and reduces neurotoxic compounds (Huang et al., 2023). Lycium ruthenicum Murray (Fan et al., 2024) increased tauroursodeoxycholic acid (TUDCA) and neurotransmitters (e.g., 5-HT, γ-aminobutyric acid), counteracting high-fat diet-induced synaptic dysfunction. Resveratrol remodeled gut microbiota, reducing TMAO as a pro-atherosclerotic metabolite associated with cognitive decline (Chen et al., 2016). Luteolin enhanced brain-derived neurotrophic factor (BDNF) and cAMP-response element binding protein (CREB) expression, promoting neurogenesis (Daily et al., 2021). Cistanche deserticola (Gao et al., 2021) and Qifu Yin (Liu et al., 2024) boost antioxidant enzymes (superoxide dismutase, glutathione peroxidase), reduced the levels of malondialdehyde (MDA) and reactive oxygen species (ROS), rescuing cognitive deficits. Saponins from Radix polygalae extent demonstrated therapeutic potential by restoring gut microbiota diversity, attenuating peripheral oxidative stress markers (e.g., lipid peroxidation products [LPO] and advanced oxidation protein products [AOPP]), and ameliorating age-associated cognitive deficits (Zeng et al., 2021). Furthermore, Shouhui Tongbian capsule exerted neuroprotective effects through dual mechanisms: suppression of lipid peroxidation and ferroptosis via modulation of Shigella and Lactobacillus populations, thereby preserving BBB integrity (Wei et al., 2025).

## Microbiota-targeted strategies for post-stroke cognitive recovery

Emerging evidence suggests that interventions targeting the microbiota represent an effective approach to alleviate PSCI. Substantial data from studies across various cognitive impairment-related pathological conditions including ischemic stroke, neurodegenerative diseases, and metabolic disorders have established robust evidence for microbial-targeted therapies (Supplementary Table S2). These strategies demonstrate the capacity to modulate gut microbiota composition, enhance beneficial metabolite production, suppress neuroinflammation, repair intestinal and blood-brain barriers, regulate neurotransmitters and neurotrophic factors, and promote synaptic plasticity. Critically, their core mechanisms of action exhibit high congruence with the pathophysiology of PSCI. Consequently, they provide essential theoretical underpinnings and potential translational avenues for developing microbiota-directed therapeutics against PSCI.

### Probiotics

Probiotics, represented by *C. butyricum, Limosilactobacillus reuteri* and *Lactobacillus plantarum* (LP), restore gut eubiosis by enriching beneficial taxa (e.g., *Bifidobacterium*, Firmicutes) while suppressing pathogens (e.g., *Enterobacteriaceae, Helicobacteriaceae*). In ischemic stroke models, butyrate produced by *C. butyricum* inhibits excessive microglial activation via Akt phosphorylation, downregulates hippocampal TNF-α/IL-1β levels, and reduces infarct volume (Sun et al., 2016a, 2020). This mechanism directly targets post-stroke neuroinflammation and is highly relevant for PSCI treatment. Furthermore, *Akkermansia muciniphila* increased SCFAs, reduced plasma endotoxins and TNF-α, IL-1β, and IL-6, and synergistically protectd blood-brain barrier integrity by upregulating the expression of intestinal Claudin-2/3 and cerebral Claudin-5, thereby improving post-stroke cognitive impairment (Li et al., 2023). It was reported *Lactobacillus rhamnosus* GG lowered serum TMAO and triglycerides in atherosclerosis models and ameliorates lipid disorders by modulating bile acid metabolism (Liang et al., 2020b). These mechanisms are highly relevant to the neuroinflammation and metabolic imbalance in PSCI. LP reduced Aβ plaques and tau protein phosphorylation while increasing levels of synaptic markers, such as PSD95 and synaptophysin (Wang et al., 2020). Moreover, LP may activate intestinal AHR signaling through TPH metabolism to counteract inflammation (Zuo et al., 2025).

### Fecal microbiota transplantation (FMT)

In stroke models, FMT reshaped the post-stroke disordered gut microbiome by introducing microbial communities from three healthy donors, which significantly increased the F/B ratio, elevated the abundance of beneficial bacteria (e.g., Akkermansiaceae, Enterobacteriaceae), and reduced pro-inflammatory bacteria (e.g., Muribaculaceae). Additionally, transplantation of young microbial communities enhanced angiogenesis and lymphatic in growth (Singh et al., 2016; Yuan et al., 2024; Chen et al., 2025a). These findings suggest that gut microbiota and their metabolites (e.g., SCFAs), inhibit neuroinflammation detrimental to cognitive recovery while promoting vascular regeneration. Although antibiotic use reduces gut microbial diversity, its rational application mitigated ischemic brain injury in mice. It was identified that fecal transplantation from antibiotic-sensitive microbiota donors significantly suppressed the trafficking of effector T cells from the gut to the leptomeninges in post-stroke mice (Benakis et al., 2016). Gut bacterial alterations further led to local Treg expansion in the small intestine and inhibition of IL-17+ γδ T effector cells, with gut-derived T cells transported to the meninges exerting neuroprotective effects. Furthermore, clinical trials have demonstrated the feasibility of fecal transplantation in modulating post-stroke outcomes (Chen et al., 2023; Zeng et al., 2023).

### Prebiotics

Prebiotics such as fructooligosaccharides (FOS), xylooligosaccharides (XOS), and yeast β-glucans modulated gut microbiota composition by increasing the abundance of *Bifidobacterium* and *Lactobacillus* while reducing *Clostridium* (Sun et al., 2019; Han et al., 2020; Xu et al., 2020b). Their mechanisms included boosting SCFA production (e.g., acetate, propionate), thereby enhancing intestinal expression of ZO-1 and occludin to reduce gut leakage and cerebral inflammatory factors. FOS improved cognitive deficits in Alzheimer's disease mice by upregulating synaptic proteins and PSD-95 expression (Sun et al., 2019). Although the models are different, the mechanism of improving synaptic plasticity has important implications for PSCI treatment. Prebiotic interventions also regulated inflammatory cytokines (e.g., IL-1β, IL-6, TNF-γ, IL-10, IL-12, IL-17α, and IL-4), inhibited microglial activation, alleviated neuroinflammation and oxidative stress, and promoted cognitive recovery [e.g., chitosan oligosaccharides (COS), oligosaccharides]. Additionally, COS demonstrated cross-system regulatory potential by improving cognitive function in hepatic encephalopathy mice via the gut-liver-brain axis (Sarkar et al., 2022; Liu et al., 2023).

### Postbiotics

**Figure 2:**
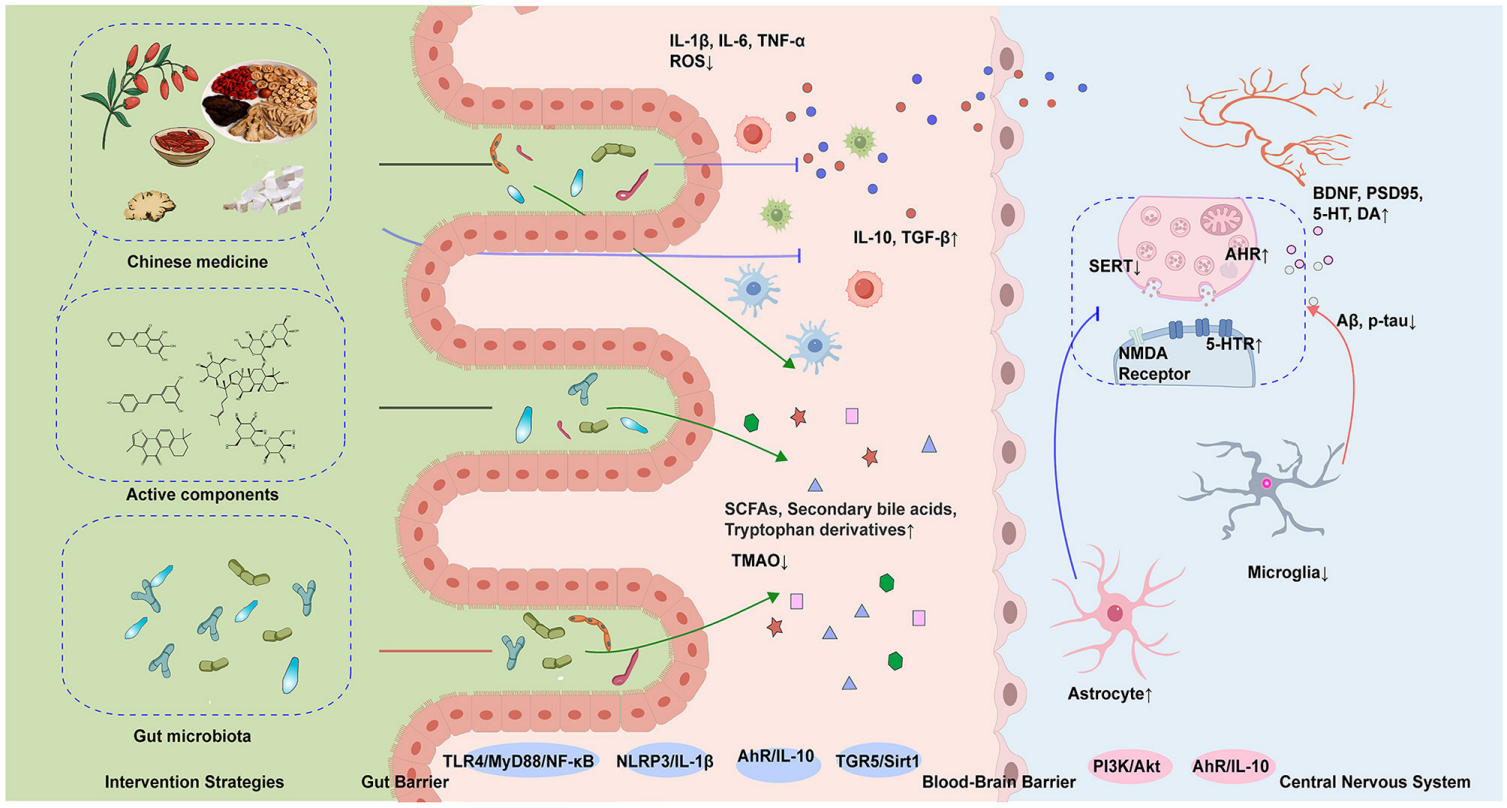
This diagram demonstrates the synergistic mechanisms of herbal medicine and microbiota in ameliorating post-stroke cognitive impairment (PSCI). Key processes include: modulation of gut microbiota structure (elevated Firmicutes/Bacteroidetes ratio) and microbial metabolites acting on the central nervous system via the gut-brain axis to enhance synaptic plasticity; dual therapeutic actions of herbal compounds involving direct suppression of cerebral TLR4/MyD88/NF-κB and NLRP3 inflammasome pathways to reduce pro-inflammatory cytokines (TNF-α, IL-1β) and neuroinflammation, alongside indirect regulation of gut microbiota composition and metabolite production. SCFAs, short-chain fatty acids; TMAO, trimethylamine n-oxide; SERT, serotonin transporter; AhR, aryl hydrocarbon receptor; NMDA, N-methyl-D-aspartate receptor; 5-HTR, 5-hydroxytryptamine receptor; BDNF, brain-derived neurotrophic factor; PSD95, postsynaptic density protein 95; DA, dopamine; TLR4, toll-like receptor 4; MyD88, myeloid differentiation primary response 88; NF-κB, nuclear factor kappa-light-chain-enhancer of activated B cells; NLRP3, NLR family pyrin domain containing 3; TGR5, G protein-coupled bile acid receptor 1; Sirt1, sirtuin 1; IL-10, interleukin-10; IL-1β, interleukin-1 beta; TGF-β, transforming growth factor beta; Aβ, β-Amyloid; p-tau, phosphorylated tau protein.

Postbiotics, defined as non-viable microbial preparations or components that confer health benefits, include inactivated probiotics and their metabolites, offering enhanced safety. Representative postbiotics such as *Bifidobacterium animalis* subsp. lactis IOBL07, Lactiplantibacillus plantarum IOB602, and IOB413 (Xiao et al., 2025) increased the abundance of Firmicutes (e.g., *Ruminococcaceae*) while reducing pathogenic bacteria (e.g., *Mucispirillum*). Promoting neurotransmitter balance and synaptic function is a key strategy for improving Post-Stroke Cognitive Impairment (PSCI). The postbiotic derived from Bifidobacterium animalis subsp. lactis IOBL07 lowered cerebral LPS levels and suppressed TLR4/NLRP3 inflammasome activity, thereby inhibiting Iba-1+ cell activation and the release of IL-6 and TNF-α. Additionally, the cell-free supernatant derived from LP directly elevated 5-HT, DA, and BDNF levels in the brain and serum, enhancing synaptic plasticity (Wu et al., 2022). In MCAO rat models, cell-free supernatant (CFS) from probiotics Lactobacillus rhamnosus UBLR-58 and Bifidobacterium breve UBBr-01 ameliorates neurological deficits in rats by improving sensorimotor performance (foot-fault, rotarod, adhesive removal, forelimb placing tests), reducing infarct volume and neuronal degradation, suppressing neuroinflammatory markers, enhancing intestinal barrier integrity (Rahman et al., 2024). This provides direct evidence of the positive effects of such strategies on neurological functional recovery in stroke models, offering robust support for PSCI applications. Figure 2 provides a schematic overview of therapeutic approaches, key mechanisms, and their implications for post-stroke cognitive impairment. Figure 2 shows the schematic diagram of the mechanism regulation of cognition impairment after stroke by Chinese medicine and its active ingredients and microbial therapy.

In summary, emerging evidence delineates a gut-brain regulatory paradigm wherein microbial metabolites, such as, SCFAs, TPH derivatives, drive PSCI by modulating microglial phenotypic reprogramming and synaptic network adaptation through neuro-immune-metabolic tripartite crosstalk. Strategic synergism between probiotic adjuvants and these metabolites enables multidimensional targeting of the microbiota-metabolite-neuroimmune axis, establishing a polypharmacological framework that surpasses the mechanistic constraints of single-pathway therapeutics.

## Synergistic applications of medicinal plants and microbial therapies

Clinical trials have demonstrated the promise of combined therapies. For instance, the Huayu Ditang Yizhi formula combined with cognitive rehabilitation (Wang et al., 2024) significantly improved MMSE and MoCA scores in post-stroke cognitive impairment (PSCI) patients, alongside increased gut microbiota diversity (Chao1 and Shannon index) and elevated neurotransmitter levels (5-HT and dopamine). Similarly, FMT from healthy donors combined with probiotics ameliorated gut dysbiosis in AD patients, reduced endotoxemia and inflammatory responses, and improved cognitive function (Fan et al., 2025). Additionally, FMT synergized with Tongnao Yizhi granules (Liu et al., 2022b) restored the Bacteroidetes/Firmicutes ratio, enhancing spatial memory in vascular cognitive impairment models. Notably, Eleutheroside E combined with FMT activated the PKA/CREB/BDNF signaling pathway via gut microbiota modulation, mitigating radiation-induced cognitive deficits (Song et al., 2022). Likewise, the combination of Corni Fructus and Limosilactobacillus reuteri concurrently modulated neuroinflammation and gut inflammation, alleviating dextran sulfate sodium (DSS)-induced colitis and cognitive dysfunction by reducing pro-inflammatory cytokines and enhancing short-chain fatty acid (SCFA) production (Lee et al., 2022). These results demonstrate that a polypharmacological approach combining phytopharmacology with microbiome-based therapeutics holds significant therapeutic potential. This strategy highlights the multi-target advantages of combined therapy in modulating the microbiota-immunity-metabolism network, paving the way for more effective interventions against cognitive impairment (Supplementary Table S3).

We therefore hypothesize that multi-target synergism and precision interventions within combined regimens will synergistically ameliorate cognitive deficits. For example, the combination of baicalin, which inhibited the TLR4/NF-κB inflammatory pathway, and *C. butyricum* (a butyrate-producing bacterium) may exert dual regulatory effects. The former directly attenuated the release of pro-inflammatory factors in the hippocampus (Song et al., 2024), whereas the latter enhanced intestinal barrier integrity via butyrate production and suppressed microglial overactivation (Sun et al., 2016a), collectively establishing an “anti-inflammatory and microbiota-metabolic” synergistic mechanism. Upon this basis, further incorporation of metabolic reprogramming strategies may enable simultaneous mitigation of oxidative damage and reinforcement of synaptic plasticity, such as schisandrin regulating brain-gut axis lipid metabolism (e.g., modulating the arachidonic acid pathway) (Zhang et al., 2022) and XOS enhancing the proliferation of butyrate-producing bacteria (Han et al., 2020). These combination methods connect local anti-inflammatory and systemic metabolic repair to form a whole, which can improve PSCI more comprehensively. Future research ought to prioritize precision microbial interventions, including dynamic “clear-rebuild” strategies. For example, phage-mediated targeting of pathogenic Enterobacteriaceae (e.g., Escherichia coli.) could reduce systemic inflammation (Chai et al., 2025). This could be followed by resveratrol administration to promote the colonization of Lactobacillus and Bifidobacterium while consolidating the balance of the flora through antioxidants. Moreover, complementary neuroregulatory approaches, such as Eleutheroside E (BDNF enhancement) coupled with Lactobacillus rhamnosus (TPH-to-5-HT conversion), could synergistically enhance gut-brain signaling through vagal pathways, collectively restoring neural-immune-metabolic homeostasis. This multi-step approach—from “pathogen inhibition” to “beneficial bacteria activation” and “neuro-flora signaling”—can systematically restore gut-brain axis.

Last and most notably, the therapeutic integration of probiotics, herbal bioactive substances, and microbial metabolites requires careful assessment of potential adverse reactions and interaction risks. First, any treatment has its appropriate target population. Current reports on adverse effects of microbial therapies predominantly focus on gastrointestinal disorders and immune-related conditions. For instance, immunocompromised individuals may develop bacteremia due to strains of Lactobacillus or *C. butyricum*, particularly in patients with immunodeficiency, gastrointestinal ulcers, or bleeding (Sada et al., 2024). Furthermore, Microbe- or microbial metabolite-drug interactions further increase the complexity of combination therapies. For instance, gut dysbiosis may enhance arsenic toxicity through two distinct mechanisms: elevating arsenic bioaccumulation and disrupting one-carbon metabolism. However, certain microbes such as *Bacteroides and Clostridium* species absorb arsenic and promote its methylation (Chi et al., 2019; Abdelsalam et al., 2020), thereby exacerbating challenges to drug stability and safety. Critically, however, adverse reactions associated with co-administration of TCM with microbes or microbial metabolites remain substantially underreported. Consequently, any inferentially derived therapeutic strategies require future validation through rigorous safety studies to ensure clinical safety.

## Summary and prospects

Despite the promising prospects of gut microbiota and TCM modulation in PSCI research, the inferred scheme assumptions still need to be verified due to the complexity before they can be truly applied to patients. The core challenge involves validating the safety and efficacy of these approaches, standardizing microbial formulations, and implementing dynamic therapeutic monitoring. Future research must prioritize three validation fronts: 1. Integrating TCM with microbiology to modulate gut microbiota through TCM principles (e.g., “warming the lungs to dispel turbidity” and “resolving phlegm stagnation while promoting descent”), while incorporating microbiological insights for comprehensive stroke interventions; 2. Multi-omics convergence of metagenomic, metabolomic, and transcriptomic data to build predictive models for microbiota-based personalized therapy; 3. Essential screening for immunosuppression, hepatic impairment, and concomitant medications, with regular monitoring of serum TMAO, hepatic enzymes, and intestinal permeability biomarkers. In summary, microbiota-metabolite modulation is driving a paradigm shift in PSCI research from “broad-spectrum interventions” to “precision restoration.” By integrating multidisciplinary technologies, future research holds the potential to achieve personalized and dynamic microbiota-based therapies, offering innovative solutions for post-stroke cognitive impairment.

## References

[B1] Aam S. Einstad M. S. Munthe-Kaas R. Lydersen S. Ihle-Hansen H. Knapskog A.-B.. (2020). Post-stroke cognitive impairment-impact of follow-up time and stroke subtype on severity and cognitive profile: the Nor-COAST study. Front Neurol. 11:699. 10.3389/fneur.2020.00699 32765406 PMC7379332

[B2] Abdelsalam N. A. Ramadan A. T. ElRakaiby M. T. Aziz R. K. (2020). Toxicomicrobiomics: the human microbiome vs. Pharmaceutical, dietary, and environmental xenobiotics. Front. Pharmacol. 11:390. 10.3389/fphar.2020.00390 32372951 PMC7179069

[B3] Agus A. Clément K. Sokol H. (2021). Gut microbiota-derived metabolites as central regulators in metabolic disorders. Gut 70, 1174–1182. 10.1136/gutjnl-2020-323071 33272977 PMC8108286

[B4] Benakis C. Brea D. Caballero S. Faraco G. Moore J. Murphy M.. (2016). Commensal microbiota affects ischemic stroke outcome by regulating intestinal γδ T cells. Nat. Med. 22, 516–523. 10.1038/nm.4068 27019327 PMC4860105

[B5] Braidy N. Grant R. (2017). Kynurenine pathway metabolism and neuroinflammatory disease. Neural. Regen. Res. 12, 39–42. 10.4103/1673-5374.198971 28250737 PMC5319230

[B6] Brunt V. E. LaRocca T. J. Bazzoni A. E. Sapinsley Z. J. Miyamoto-Ditmon J. Gioscia-Ryan R. A.. (2020). The gut microbiome–derived metabolite trimethylamine N-oxide modulates neuroinflammation and cognitive function with aging. GeroScience 43, 377–394. 10.1007/s11357-020-00257-2 32862276 PMC8050157

[B7] Chai J. Sun H. Schwarz S. Huang Y. Xie S. Xu Q.. (2025). Isolation, characterization, and application of the novel polyvalent bacteriophage vB_EcoM_XAM237 against pathogenic escherichia coli. Vet. Res. 56:90. 10.1186/s13567-025-01514-y 40275417 PMC12023626

[B8] Chen A. Teng C. Wei J. Wu X. Zhang H. Chen P.. (2025a). Gut microbial dysbiosis exacerbates long-term cognitive impairments by promoting intestinal dysfunction and neuroinflammation following neonatal hypoxia-ischemia. Gut Microbes 17:2471015. 10.1080/19490976.2025.2471015 40008452 PMC11866968

[B9] Chen C. Xiao Q. Wen Z. Gong F. Zhan H. Liu J.. (2025b). Gut microbiome-derived indole-3-carboxaldehyde regulates stress vulnerability in chronic restraint stress by activating aryl hydrocarbon receptors. Pharmacol. Res. 213:107654. 10.1016/j.phrs.2025.107654 39946793

[B10] Chen G. Gao C. Yan Y. Wang T. Luo C. Zhang M.. (2020). Inhibiting ER stress weakens neuronal pyroptosis in a mouse acute hemorrhagic stroke model. Mol. Neurobiol. 57, 5324–5335. 10.1007/s12035-020-02097-9 32880859

[B11] Chen M. Yi L. Zhang Y. Zhou X. Ran L. Yang J.. (2016). Resveratrol Attenuates Trimethylamine-N-Oxide (TMAO)-induced atherosclerosis by regulating TMAO synthesis and bile acid metabolism via remodeling of the gut microbiota. Mbio 7, e02210–2215. 10.1128/mBio.02210-15 27048804 PMC4817264

[B12] Chen R. Wu P. Cai Z. Fang Y. Zhou H. Lasanajak Y.. (2019a). Puerariae Lobatae Radix with chuanxiong Rhizoma for treatment of cerebral ischemic stroke by remodeling gut microbiota to regulate the brain-gut barriers. J. Nutr. Biochem. 65, 101–114. 10.1016/j.jnutbio.2018.12.004 30710886

[B13] Chen R. Xu Y. Wu P. Zhou H. Lasanajak Y. Fang Y.. (2019b). Transplantation of fecal microbiota rich in short chain fatty acids and butyric acid treat cerebral ischemic stroke by regulating gut microbiota. Pharmacol. Res. 148:104403. 10.1016/j.phrs.2019.104403 31425750

[B14] Chen T. Noto D. Hoshino Y. Mizuno M. Miyake S. (2019c). Butyrate suppresses demyelination and enhances remyelination. J. Neuroinflammation. 16:165. 10.1186/s12974-019-1552-y 31399117 PMC6688239

[B15] Chen T. Wang L. Xie G. Kristal B. S. Zheng X. Sun T.. (2024). Serum bile acids improve prediction of alzheimer's progression in a sex-dependent manner. Adv Sci. 11:e2306576. 10.1002/advs.202306576 38093507 PMC10916590

[B16] Chen X. Zhang W. Lin Z. Zheng C. Chen S. Zhou H.. (2023). Preliminary evidence for developing safe and efficient fecal microbiota transplantation as potential treatment for aged related cognitive impairments. Front. Cell Infect Microbiol. 13:1103189. 10.3389/fcimb.2023.1103189 37113132 PMC10127103

[B17] Chen Y. Liang J. Ouyang F. Chen X. Lu T. Jiang Z.. (2019d). Persistence of gut microbiota dysbiosis and chronic systemic inflammation after cerebral infarction in cynomolgus monkeys. Front. Neurol. 10:661. 10.3389/fneur.2019.00661 31316450 PMC6611357

[B18] Chi L. Xue J. Tu P. Lai Y. Ru H. Lu K. (2019). Gut microbiome disruption altered the biotransformation and liver toxicity of arsenic in mice. Arch. Toxicol. 93, 25–35. 10.1007/s00204-018-2332-7 30357543 PMC7727877

[B19] Cho B. P. H. Nannoni S. Harshfield E. L. Tozer D. Gräf S. Bell S.. (2021). NOTCH3 variants are more common than expected in the general population and associated with stroke and vascular dementia: an analysis of 200 000 participants. J. Neurol. Neurosurg. Psychiatry 92, 694–701. 10.1136/jnnp-2020-325838 33712516 PMC8223663

[B20] Cipollini V. Troili F. Giubilei F. (2019). Emerging biomarkers in vascular cognitive impairment and dementia: from pathophysiological pathways to clinical application. Int. J. Mol. Sci. 20:2812. 10.3390/ijms20112812 31181792 PMC6600494

[B21] Cogo A. Mangin G. Maïer B. Callebert J. Mazighi M. Chabriat H.. (2021). Increased serum QUIN/KYNA is a reliable biomarker of post-stroke cognitive decline. Mol. Neurodegener. 16:7. 10.1186/s13024-020-00421-4 33588894 PMC7885563

[B22] Collins S. L. Stine J. G. Bisanz J. E. Okafor C. D. Patterson A. D. (2023). Bile acids and the gut microbiota: metabolic interactions and impacts on disease. Nat. Rev. Microbiol. 21, 236–247. 10.1038/s41579-022-00805-x 36253479 PMC12536349

[B23] Daily J. W. Kang S. Park S. (2021). Protection against Alzheimer's disease by luteolin: Role of brain glucose regulation, anti-inflammatory activity, and the gut microbiota-liver-brain axis. Biofactors 47, 218–231. 10.1002/biof.1703 33347668

[B24] Dantas Machado A. C. Ramos S. F. Gauglitz J. M. Fassler A.-M. Petras D. Aksenov A. A.. (2023). Portosystemic shunt placement reveals blood signatures for the development of hepatic encephalopathy through mass spectrometry. Nat. Commun. 14:5303. 10.1038/s41467-023-40741-9 37652904 PMC10471626

[B25] Fan S. Wang T. Wen G. (2025). Clinical efficacy of fecal microbiota transplantation combined with probiotics for moderate elderly AD patients. Chin. J. Geriatr. Heart Brain Vessel Dis. 27, 192–196.

[B26] Fan X. Dong W. Huang Y. Shu Y. Yan Y. Mi J.. (2024). Aqueous extract of lycium ruthenicum murray attenuates neuroinflammation in C57BL/6J mice induced by high-fat and high-fructose diet through regulating gut microbiota and bile acid metabolism. Foods 13:3812. 10.3390/foods13233812 39682885 PMC11640740

[B27] Fu J. Li J. Sun Y. Liu S. Song F. Liu Z. (2023). In-depth investigation of the mechanisms of Schisandra chinensis polysaccharide mitigating Alzheimer's disease rat via gut microbiota and feces metabolomics. Int. J. Biol. Macromol. 232:123488. 10.1016/j.ijbiomac.2023.123488 36731694

[B28] Fu Y. Hou X. Feng Z. Feng H. Li L. (2024). Research progress in the relationship between gut microbiota metabolite trimethylamine N-oxide and ischemic stroke. Zhong Nan Xue Xue Bao, Yi Xue Ban. J. Cent. South Univ. Med. Sci. 49, 447–456. 10.11817/j.issn.1672-7347.2024.230427 38970519 PMC11208405

[B29] Gallucci L. Sperber C. Guggisberg A. G. Kaller C. P. Heldner M. R. Monsch A. U.. (2024). Post-stroke cognitive impairment remains highly prevalent and disabling despite state-of-the-art stroke treatment. Int. J. Stroke 19, 888–897. 10.1177/17474930241238637 38425239

[B30] Gao Y. Li B. Liu H. Tian Y. Gu C. Du X.. (2021). Cistanche deserticola polysaccharides alleviate cognitive decline in aging model mice by restoring the gut microbiota-brain axis. Aging 13, 15320–15335. 10.18632/aging.203090 34081627 PMC8221331

[B31] Govindarajulu M. Pinky P. D. Steinke I. Bloemer J. Ramesh S. Kariharan T.. (2020). Gut metabolite TMAO induces synaptic plasticity deficits by promoting endoplasmic reticulum stress. Front. Mol. Neurosci. 13:138. 10.3389/fnmol.2020.00138 32903435 PMC7437142

[B32] Gu X. Zhou J. Zhou Y. Wang H. Si N. Ren W.. (2021). Huanglian Jiedu decoction remodels the periphery microenvironment to inhibit Alzheimer's disease progression based on the “brain-gut” axis through multiple integrated omics. Alzheimers Res. Ther. 13:44. 10.1186/s13195-021-00779-7 33579351 PMC7881564

[B33] Han D. Li Z. Liu T. Yang N. Li Y. He J.. (2020). Prebiotics Regulation of intestinal microbiota attenuates cognitive dysfunction induced by surgery stimulation in APP/PS1 mice. Aging Dis. 11, 1029–1045. 10.14336/AD.2020.0106 33014520 PMC7505279

[B34] Hertelendy P. Toldi J. Fülöp F. Vécsei L. (2018). Ischemic stroke and kynurenines: medicinal chemistry aspects. Curr. Med. Chem. 25, 5945–5957. 10.2174/0929867325666180313113411 29532751

[B35] Hu K. Zhou Z. Li H. Xiao J. Shen Y. Ding K.. (2025). Regulation of histidine metabolism by Lactobacillus Reuteri mediates the pathogenesis and treatment of ischemic stroke. Acta. Pharm. Sin B. 15, 239–255. 10.1016/j.apsb.2024.10.003 40041923 PMC11873608

[B36] Huang H.-S. Lin Y.-E. Panyod S. Chen R.-A. Lin Y.-C. Chai L. M. X.. (2023). Anti-depressive-like and cognitive impairment alleviation effects of Gastrodia elata Blume water extract is related to gut microbiome remodeling in ApoE-/- mice exposed to unpredictable chronic mild stress. J. Ethnopharmacol. 302:115872. 10.1016/j.jep.2022.115872 36343797

[B37] Iadecola C. Buckwalter M. S. Anrather J. (2020). Immune responses to stroke: mechanisms, modulation, and therapeutic potential. J. Clin. Invest 130, 2777–2788. 10.1172/JCI135530 32391806 PMC7260029

[B38] Jia M. Fan Y. Ma Q. Yang D. Wang Y. He X.. (2024). Gut microbiota dysbiosis promotes cognitive impairment via bile acid metabolism in major depressive disorder. Transl. Psychiatry 14:503. 10.1038/s41398-024-03211-4 39719433 PMC11668851

[B39] Keitel V. Görg B. Bidmon H. J. Zemtsova I. Spomer L. Zilles K.. (2010). The bile acid receptor TGR5 (Gpbar-1) acts as a neurosteroid receptor in brain. Glia 58, 1794–1805. 10.1002/glia.21049 20665558

[B40] Keller J. Gomez R. Williams G. Lembke A. Lazzeroni L. Murphy G. M.. (2017). HPA axis in major depression: cortisol, clinical symptomatology and genetic variation predict cognition. Mol. Psychiatry 22, 527–536. 10.1038/mp.2016.120 27528460 PMC5313380

[B41] Kong Q. Chen Q. Mao X. Wang G. Zhao J. Zhang H.. (2022). Bifidobacterium longum CCFM1077 ameliorated neurotransmitter disorder and neuroinflammation closely linked to regulation in the kynurenine pathway of autistic-like rats. Nutrients 14:1615. 10.3390/nu14081615 35458177 PMC9031594

[B42] Lee H. L. Kim J. M. Moon J. H. Kim M. J. Jeong H. R. Go M. J.. (2022). Anti-amnesic effect of synbiotic supplementation containing corni fructus and limosilactobacillus reuteri in DSS-induced colitis mice. Int. J. Mol. Sci. 24:90. 10.3390/ijms24010090 36613533 PMC9820465

[B43] Li C. Wang X. Yan J. Cheng F. Ma X. Chen C.. (2020). Cholic acid protects *In vitro* neurovascular units against oxygen and glucose deprivation-induced injury through the BDNF-TrkB signaling pathway. Oxid. Med. Cell. Longevity 2020:1201624. 10.1155/2020/1201624 33101581 PMC7576336

[B44] Li N. Tan S. Wang Y. Deng J. Wang N. Zhu S.. (2023). Akkermansia muciniphila supplementation prevents cognitive impairment in sleep-deprived mice by modulating microglial engulfment of synapses. Gut Microbes 15:2252764. 10.1080/19490976.2023.2252764 37671803 PMC10484034

[B45] Li Y. Zhang J. Lei Y. Chang M. Xu J. Tang S. (2025). Multi-omics approaches reveal the therapeutic mechanism of Naoxintong capsule against ischemic stroke. J Ethnopharmacol. 343:119435. 10.1016/j.jep.2025.119435 39909118

[B46] Liang H. Matei N. McBride D. W. Xu Y. Tang J. Luo B.. (2020a). Activation of TGR5 protects blood brain barrier via the BRCA1/Sirt1 pathway after middle cerebral artery occlusion in rats. J. Biomed. Sci. 27:61. 10.1186/s12929-020-00656-9 32381096 PMC7206796

[B47] Liang X. Zhang Z. Lv Y. Tong L. Liu T. Yi H.. (2020b). Reduction of intestinal trimethylamine by probiotics ameliorated lipid metabolic disorders associated with atherosclerosis. Nutrition 79–80, 110941. 10.1016/j.nut.2020.110941 32858376

[B48] Liao M. Long X. Chen Y. An J. Huang W. Xu X.. (2025). PARP9 exacerbates apoptosis and neuroinflammation via the PI3K pathway in the thalamus and hippocampus and cognitive decline after cortical infarction. J. Neuroinflammation 22:43. 10.1186/s12974-025-03374-x 39980030 PMC11844078

[B49] Liu H. Li R. Su K. Yuan J. Li Q. Feng X. (2022a). Latest research on the neuroprotective mechanism of short-chain fatty acids in stroke and its relation with post-stroke cognitive impairment. Chin. Gen. Pract. 25, 380–386.

[B50] Liu H. Yan X. Zhang Q. Meng T. Liu J. Chang (2022b). Effects of Tongnao Yizhi granules combined with fecal microbiota transplantation on learning and memory ability and gut microbiota of rats with vascular cognitive impairment. Modern Journal of Integrated Traditional Chinese and Western Medicine 31, 2786–2792+2862.

[B51] Liu J. Li H. Gong T. Chen W. Mao S. Kong Y.. (2020a). Anti-neuroinflammatory Effect of Short-Chain Fatty Acid Acetate against Alzheimer's Disease via Upregulating GPR41 and Inhibiting ERK/JNK/NF-κB. J. Agric. Food Chem. 68, 7152–7161. 10.1021/acs.jafc.0c02807 32583667

[B52] Liu J. Zhang T. Wang Y. Si C. Wang X. Wang R.-T.. (2020b). Baicalin ameliorates neuropathology in repeated cerebral ischemia-reperfusion injury model mice by remodeling the gut microbiota. Aging 12, 3791–3806. 10.18632/aging.102846 32084011 PMC7066900

[B53] Liu P. Li H. Xu H. Gong J. Jiang M. Qian J.. (2023). Chitooligosaccharides Attenuated Hepatic Encephalopathy in Mice through Stabilizing Gut-Liver-Brain Disturbance. Mol Nutr Food Res 67:e2200158. 10.1002/mnfr.202370001 36281912

[B54] Liu S. Zhang Q. Zhao F. Deng F. Wang Y. (2024). Regulating effect of Qifu Yin on intestinal microbiota in mice with memory impairment induced by scopolamine hydrobromide. J. Ethnopharmacol . 333:118445. 10.1016/j.jep.2024.118445 38851472

[B55] Liu Y. Kong C. Gong L. Zhang X. Zhu Y. Wang H.. (2020c). The Association of Post-Stroke Cognitive Impairment and Gut Microbiota and its Corresponding Metabolites. J Alzheimers Dis 73, 1455–1466. 10.3233/JAD-191066 31929168

[B56] MahmoudianDehkordi S. Arnold M. Nho K. Ahmad S. Jia W. Xie G.. (2019). Altered bile acid profile associates with cognitive impairment in alzheimer's disease – an emerging role for gut microbiome - PMC. Alzheimer. Demen. 15, 76–92. 10.1016/j.jalz.2018.07.217 30337151 PMC6487485

[B57] MAO Z. ZHANG J. GUO L. WANG X. ZHU Z. MIAO M. (2024). Therapeutic approaches targeting the gut microbiota in ischemic stroke: current advances and future directions. Biosci. Microbiota. Food Health 43, 321–328. 10.12938/bmfh.2024-022 39364121 PMC11444859

[B58] Martin C. R. Osadchiy V. Kalani A. Mayer E. A. (2018). The brain-gut-microbiome axis. Cell Mol. Gastroenterol. Hepatol. 6, 133–148. 10.1016/j.jcmgh.2018.04.003 30023410 PMC6047317

[B59] Meng F. Li N. Li D. Song B. Li L. (2019). The presence of elevated circulating trimethylamine N-oxide exaggerates postoperative cognitive dysfunction in aged rats. Behav. Brain Res. 368:111902. 10.1016/j.bbr.2019.111902 30980850

[B60] O'Riordan K. J. Collins M. K. Moloney G. M. Knox E. G. Aburto M. R. Fülling C.. (2022). Short chain fatty acids: Microbial metabolites for gut-brain axis signalling. Mol. Cell Endocrinol. 546:111572. 10.1016/j.mce.2022.111572 35066114

[B61] Park J. K. Lee K. J. Kim J. Y. Kim H. (2021). The association of blood-based inflammatory factors IL-1β, TGF-β and CRP with cognitive function in alzheimer's disease and mild cognitive impairment. Psychiatry Investig. 18, 11–18. 10.30773/pi.2020.0205 33561929 PMC7897864

[B62] Qi Y. Wang X. Zhang Y. Leng Y. Liu X. Wang X.. (2023). Walnut-derived peptide improves cognitive impairment in colitis mice induced by dextran sodium sulfate via the microbiota-gut-brain axis (MGBA). J. Agric. Food Chem. 71, 19501–19515. 10.1021/acs.jafc.3c04807 38039336

[B63] Qian X. Li Q. Zhu H. Chen Y. Lin G. Zhang H.. (2024). Bifidobacteria with indole-3-lactic acid-producing capacity exhibit psychobiotic potential via reducing neuroinflammation. Cell Rep. Med. 5:101798. 10.1016/j.xcrm.2024.101798 39471819 PMC11604549

[B64] Quinn T. J. Richard E. Teuschl Y. Gattringer T. Hafdi M. O'Brien J. T.. (2021). European Stroke Organisation and European Academy of Neurology joint guidelines on post-stroke cognitive impairment. Eur. Stroke J. 6, I–XXXVIII. 10.1177/23969873211042192 34746430 PMC8564156

[B65] Rahman Z. Padhy H. P. Dandekar M. P. (2024). Cell-free supernatant of lactobacillus rhamnosus and bifidobacterium breve ameliorates ischemic stroke-generated neurological deficits in rats. Probiotics Antimicrob. Proteins 10.1007/s12602-024-10256-w 38656733

[B66] Rodrigues C. M. P. Spellman S. R. Solá S. Grande A. W. Linehan-Stieers C. Low W. C.. (2002). Neuroprotection by a bile acid in an acute stroke model in the rat. J. Cereb. Blood Flow Metab. 22, 463–471. 10.1097/00004647-200204000-00010 11919517

[B67] Romano K. A. Vivas E. I. Amador-Noguez D. Rey F. E. (2015). Intestinal microbiota composition modulates choline bioavailability from diet and accumulation of the proatherogenic metabolite trimethylamine-N-oxide. mBio 6, e02481–e02414. 10.1128/mBio.02481-14 25784704 PMC4453578

[B68] Rost N. S. Brodtmann A. Pase M. P. van Veluw S. J. Biffi A. Duering M.. (2022). Post-stroke cognitive impairment and dementia. Circ. Res. 130, 1252–1271. 10.1161/CIRCRESAHA.122.319951 35420911

[B69] Roth W. Zadeh K. Vekariya R. Ge Y. Mohamadzadeh M. (2021). Tryptophan metabolism and gut-brain homeostasis. Int. J. Mol. Sci .22:2973. 10.3390/ijms22062973 33804088 PMC8000752

[B70] Rutsch A. Kantsjö J. B. Ronchi F. (2020). The gut-brain axis: how microbiota and host inflammasome influence brain physiology and pathology. Front. Immunol. 11:604179. 10.3389/fimmu.2020.604179 33362788 PMC7758428

[B71] Sada R. M. Matsuo H. Motooka D. Kutsuna S. Hamaguchi S. Yamamoto G.. (2024). *C. butyricum bacteremia* associated with probiotic use, Japan. Emerg. Infect Dis. 30, 665–671. 10.3201/eid3004.231633 38413242 PMC10977840

[B72] Sandvig H. V. Aam S. Alme K. N. Lydersen S. Magne Ueland P. Ulvik A.. (2024). Neopterin, kynurenine metabolites, and indexes related to vitamin B6 are associated with post-stroke cognitive impairment: the Nor-COAST study. Brain Behav Immun. 118, 167–177. 10.1016/j.bbi.2024.02.030 38428649

[B73] Sarkar S. R. Mazumder P. M. Banerjee S. (2022). Oligosaccharide and flavanoid mediated prebiotic interventions to treat gut dysbiosis associated cognitive decline. J Neuroimmune Pharmacol. 17, 94–110. 10.1007/s11481-021-10041-4 35043295

[B74] Silva Y. P. Bernardi A. Frozza R. L. (2020). The role of short-chain fatty acids from gut microbiota in gut-brain communication. Front Endocrinol 11:25. 10.3389/fendo.2020.00025 32082260 PMC7005631

[B75] Singh V. Roth S. Llovera G. Sadler R. Garzetti D. Stecher B.. (2016). Microbiota dysbiosis controls the neuroinflammatory response after stroke. J. Neurosci. 36, 7428–7440. 10.1523/JNEUROSCI.1114-16.2016 27413153 PMC6705544

[B76] Song C. Duan F. Ju T. Qin Y. Zeng D. Shan S.. (2022). Eleutheroside E supplementation prevents radiation-induced cognitive impairment and activates PKA signaling via gut microbiota. Commun. Biol. 5:680. 10.1038/s42003-022-03602-7 35804021 PMC9270490

[B77] Song J. Li M. Kang N. Jin W. Xiao Y. Li Z.. (2024). Baicalein ameliorates cognitive impairment of vascular dementia rats via suppressing neuroinflammation and regulating intestinal microbiota. Brain Res. Bull 208:110888. 10.1016/j.brainresbull.2024.110888 38295883

[B78] Su H. Fan S. Zhang L. Qi H. (2021). TMAO Aggregates neurological damage following ischemic stroke by promoting reactive astrocytosis and glial scar formation via the Smurf2/ALK5 axis. Front. Cell Neurosci. 15:569424. 10.3389/fncel.2021.569424 33815059 PMC8012716

[B79] Sun J. Liu S. Ling Z. Wang F. Ling Y. Gong T.. (2019). Fructooligosaccharides ameliorating cognitive deficits and neurodegeneration in APP/PS1 transgenic mice through modulating gut microbiota. J. Agric. Food Chem. 67, 3006–3017. 10.1021/acs.jafc.8b07313 30816709

[B80] Sun J. Wang F. Ling Z. Yu X. Chen W. Li H.. (2016a). *C. butyricum attenuates* cerebral ischemia/reperfusion injury in diabetic mice via modulation of gut microbiota. Brain Res. 1642, 180–188. 10.1016/j.brainres.2016.03.042 27037183

[B81] Sun J. Xu J. Yang B. Chen K. Kong Y. Fang N.. (2020). Effect of *C. butyricum* against microglia-mediated neuroinflammation in alzheimer's disease via regulating gut microbiota and metabolites butyrate. Mol. Nutr. Food Res. 64:e1900636. 10.1002/mnfr.201900636 31835282

[B82] Sun P. Wang M. Li Z. Wei J. Liu F. Zheng W.. (2022). Eucommiae cortex polysaccharides mitigate obesogenic diet-induced cognitive and social dysfunction via modulation of gut microbiota and tryptophan metabolism. Theranostics 12, 3637–3655. 10.7150/thno.72756 35664075 PMC9131264

[B83] Sun X. Jiao X. Ma Y. Liu Y. Zhang L. He Y.. (2016b). Trimethylamine N-oxide induces inflammation and endothelial dysfunction in human umbilical vein endothelial cells via activating ROS-TXNIP-NLRP3 inflammasome. Biochem. Biophys. Res. Commun. 481, 63–70. 10.1016/j.bbrc.2016.11.017 27833015

[B84] Tack R. W. P. Amboni C. van Nuijs D. Pekna M. Vergouwen M. D. I. Rinkel G. J. E.. (2025). Inflammation, anti-inflammatory interventions, and post-stroke cognitive impairment: a systematic review and meta-analysis of human and animal studies. Transl. Stroke Res. 16, 535–546. 10.1007/s12975-023-01218-5 38012509 PMC11976800

[B85] Tu R. Xia J. (2024). Stroke and vascular cognitive impairment: the role of intestinal microbiota metabolite TMAO. CNS Neurol. Disord. Drug Targets 23, 102–121. 10.2174/1871527322666230203140805 36740795

[B86] Wang H. Zhang M. Li J. Liang J. Yang M. Xia G.. (2022). Gut microbiota is causally associated with poststroke cognitive impairment through lipopolysaccharide and butyrate. J. Neuroinflammation 19:76. 10.1186/s12974-022-02435-9 35379265 PMC8981610

[B87] Wang J. Cheng W. Cheng G. (2024). Clinical efficacy of huayu ditan yizhi formula combined with cognitive rehabilitationtraining on post-stroke cognitive impairment and its effect on gut microbiota diversity. Inform. Trad. Chin. Med. 41, 47–51+57.

[B88] Wang Q.-J. Shen Y.-E. Wang X. Fu S. Zhang X. Zhang Y.-N.. (2020). Concomitant memantine and Lactobacillus plantarum treatment attenuates cognitive impairments in APP/PS1 mice. Aging 12, 628–649. 10.18632/aging.102645 31907339 PMC6977692

[B89] Wang X. Chen A. Wu H. Ye M. Cheng H. Jiang X.. (2016). Enriched environment improves post-stroke cognitive impairment in mice by potential regulation of acetylation homeostasis in cholinergic circuits. Brain Res. 1650, 232–242. 10.1016/j.brainres.2016.09.018 27637156

[B90] Wei F. Zhou J. Pan L. Shen M. Niu D. Zeng Z.. (2025). Integrative microbiomics, proteomics and lipidomics studies unraveled the preventive mechanism of Shouhui Tongbian Capsules on cerebral ischemic stroke injury. J. Ethnopharmacol. 337:118874. 10.1016/j.jep.2024.118874 39362332

[B91] Wu Y. Wang Y. Hu A. Shu X. Huang W. Liu J.. (2022). Lactobacillus plantarum-derived postbiotics prevent Salmonella-induced neurological dysfunctions by modulating gut-brain axis in mice. Front. Nutr. 9:946096. 10.3389/fnut.2022.946096 35967771 PMC9365972

[B92] Xiao L. Tang L. Song X. Zhang Y. Han X. Lv H.. (2025). Postbiotics regulate intestinal microbiota and reduce Aβ deposition in the brain to improve cognitive impairment in AD rats. Food Sci. 46, 182–193.

[B93] Xing C. Huang X. Wang D. Yu D. Hou S. Cui H.. (2023). Roles of bile acids signaling in neuromodulation under physiological and pathological conditions. Cell Biosci. 13:106. 10.1186/s13578-023-01053-z 37308953 PMC10258966

[B94] Xu H. Chai S. Wang Y. Wang J. Xiao D. Li J.. (2020a). Molecular and clinical characterization of PARP9 in gliomas: a potential immunotherapeutic target. CNS Neurosci. Ther. 26, 804–814. 10.1111/cns.13380 32678519 PMC7366751

[B95] Xu M. Mo X. Huang H. Chen X. Liu H. Peng Z.. (2020b). Yeast β-glucan alleviates cognitive deficit by regulating gut microbiota and metabolites in Aβ1-42-induced AD-like mice. Int. J. Biol. Macromol. 161, 258–270. 10.1016/j.ijbiomac.2020.05.180 32522544

[B96] Yang J. Ran M. Li H. Lin Y. Ma K. Yang Y.. (2022). New insight into neurological degeneration: Inflammatory cytokines and blood–brain barrier. Front. Mol. Neurosci. 15:1013933. 10.3389/fnmol.2022.1013933 36353359 PMC9637688

[B97] Yang P. Fan X. Liu M. Liu J. Cao L. Wang Z.. (2024). Effects of epimedium total flavone capsules on post-stroke cognitive impairment in rats. 中国中药杂志 49, 2262–2272. 10.19540/j.cnki.cjcmm.20240118.104 38812240

[B98] Yuan Y. Li L. Wang J. Myagmar B.-O. Gao Y. Wang H.. (2024). Gut microbiota-derived acetate promotes long-term recovery through angiogenesis guided by lymphatic ingrowth in older adults with stroke. Front. Neurosci. 18:1398913. 10.3389/fnins.2024.1398913 39371609 PMC11450648

[B99] Zeng W. Wu A. G. Zhou X.-G. Khan I. Zhang R. L. Lo H. H.. (2021). Saponins isolated from Radix polygalae extent lifespan by modulating complement C3 and gut microbiota. Pharmacol. Res. 170:105697. 10.1016/j.phrs.2021.105697 34062240

[B100] Zeng X. Li J. Shan W. Lai Z. Zuo Z. (2023). Gut microbiota of old mice worsens neurological outcome after brain ischemia via increased valeric acid and IL-17 in the blood. Microbiome 11:204. 10.1186/s40168-023-01648-1 37697393 PMC10496352

[B101] Zhan C. Huang Q. Zhang T. Yuan J. Yuan L. Su Z.. (2023). A study on the mechanism of the Wenfei Jiangzhuo recipe in the treatment of vascular dementia based on the microbial-gut-brain axis theory. Clin. J. Chin. Med. 15, 77–82.

[B102] Zhang C. Zhang Y. Zhao T. Mou T. Jing W. Chen J.. (2022). Schisandrin alleviates the cognitive impairment in rats with Alzheimer's disease by altering the gut microbiota composition to modulate the levels of endogenous metabolites in the plasma, brain, and feces. Front Pharmacol. 13:888726. 10.3389/fphar.2022.888726 36176456 PMC9514097

[B103] Zhang F. Deng Y. Wang H. Fu J. Wu G. Duan Z.. (2024). Gut microbiota-mediated ursodeoxycholic acids regulate the inflammation of microglia through TGR5 signaling after MCAO. Brain Behav. Immun. 115, 667–679. 10.1016/j.bbi.2023.11.021 37989444

[B104] Zhang J. Wang L. Cai J. Lei A. Liu C. Lin R.. (2021a). Gut microbial metabolite TMAO portends prognosis in acute ischemic stroke. J. Neuroimmunol. 354:577526. 10.1016/j.jneuroim.2021.577526 33647820

[B105] Zhang X. Yuan M. Yang S. Chen X. Wu J. Wen M.. (2021b). Enriched environment improves post-stroke cognitive impairment and inhibits neuroinflammation and oxidative stress by activating Nrf2-ARE pathway. Int. J. Neurosci. 131, 641–649. 10.1080/00207454.2020.1797722 32677581

[B106] Zheng D. Liwinski T. Elinav E. (2020). Interaction between microbiota and immunity in health and disease. Cell Res. 30, 492–506. 10.1038/s41422-020-0332-7 32433595 PMC7264227

[B107] Zhou Z. Xu N. Matei N. McBride D. W. Ding Y. Liang H.. (2021). Sodium butyrate attenuated neuronal apoptosis via GPR41/Gβγ/PI3K/Akt pathway after MCAO in rats. J. Cereb. Blood Flow Metab. 41, 267–281. 10.1177/0271678X20910533 32151222 PMC8370004

[B108] Zhu C. Li G. Lv Z. Li J. Wang X. Kang J.. (2020). Association of plasma trimethylamine-N-oxide levels with post-stroke cognitive impairment: a 1-year longitudinal study. Neurol. Sci. 41, 57–63. 10.1007/s10072-019-04040-w 31420758

[B109] Zuo X. Zhang T. Dong X. Liu J. Liu Y. (2025). Identification of the key tryptophan metabolic characteristics of lactiplantibacillus plantarum for aryl hydrocarbon receptor activation and ulcerative colitis alleviation. Food Res. Int. 203:115766. 10.1016/j.foodres.2025.115766 40022314

