# Supplementary material for: Synergistic therapy of Chinese herbal medicine and gut microbiota modulation for post-stroke cognitive recovery: focus on microbial metabolite and immunoinflammation
Source: Front Microbiol. 2025 Aug 14;16:1623843. doi: 10.3389/fmicb.2025.1623843 (PMC12391124; doi:10.3389/fmicb.2025.1623843)
Supplement: Supplementary file 1 [file Table_1.docx]

Supplementary Material

**Table S1 Effects of Chinese medicine and components on gut microbiota and Index changes associated with PSCI.**

| Drugs | Species/Strain | Diseases | Effects on related biomarkers | Intestinal microbiota modulation | Conclusion | Reference |
| --- | --- | --- | --- | --- | --- | --- |
| Baicalin | Male Sprague-Dawley rats | vascular dementia (VD) | Hippocampal Tumor Necrosis Factor-alpha (TNF-α), IL-6 (Interleukin-6), Interleukin-1 beta (IL-1β) , Toll-Like Receptor 4 (TLR4), Myeloid Differentiation Factor 88 (MyD88), and Nuclear Factor Kappa B (NF-κB)**↓,** and the number of neuroglial cells **↓**. | Shannon index **↑**; Bacteroidetes, *Blautia*, and *Eubacterium* **↑**; Firmicutes *,Lactobacillus* and *Clostridium* **↓** | Baicalin ameliorated cognitive impairment in VD rats, alleviated hippocampal inflammation. | (Song et al., 2024) |
| *Lycium ruthenicum Murray*（LRE） | male C57BL/6J mice | Neuroinflammation induced by high-fat and high-fructose diet (HFFD) | TNF-α, IL-6, and IL-1β in the cerebral cortex and hippocampus ↓, while tauroursodeoxycholic acid and taurocholic acid in the hippocampus and cerebral cortex ↑. | *Streptococcus and* *probiotics* ↑; *Helicobacter* and *Clostridium_*XIVa↓ | LRE alleviated HFFD-induced cognitive dysfunction by modulating intestinal microbiota and promoting the synthesis of neuroprotective BAs. | (Fan et al., 2024) |
| Schisandrin | male SD rats | Alzheimer’s disease (AD) | Zonula Occludens-1 (ZO-1) and occludin in the colon ↑, IL-6, TNF-α, and IL-1β ↓, while acetate, propionate, and butyrate **↑**. | Firmicutes, *lactobacillus*, Ruminococcaceae*_UCG-005*, *clostridium* ↑; Bacteroides , *escherichia-Shigella*, *enterorhabdus, bacteroides,* and *alloprevotella*↓ | The mechanisms involves microbial metabolic pathways, thereby intervening in the gut-brain axis at the metabolic level. | (Zhang et al., 2022; Fu et al., 2023) |
| *Panax notoginsen*g saponins (PNS) | Male Sprague–Dawley rats，OGD/R-PC12 cells | Ischemic stroke (IS) | TNF-α, IL-1β, and IL-6 in the brain and cells ↓, while histidine in the serum and brain ↑. | Firmicutes, *Lactobacillus* ↑; Proteobacteria↓ | PNS could promote histidine synthesis in gut by enhancing L.Reu proportion, thereby increasing intracerebral histidine through peripheral pathway,thereby improving stroke. | (Hu et al., 2025) |
| Naoximintong capsule (NXT) | Male C57BL/6J mice | IS | Intracerebral Lipopolysaccharide (LPS), IL-1β, Interleukin-17 beta (IL-17A), and IL-6 ↓, intestinal ZO-1, Mucin 2 ↑, and serum serine, lysine, and proline ↑. | Firmicutes / Bacteroidetes (F/B) ratio ↑; Sutterellaceae*, Ihubacter, Parasutterella, Bacteroides thetaiotaomicron, Massiliensis,* and *excrementihominis* ↑; Sutterellaceae, *Parasutterella*, and *excrementihominis* ↑ | NXT alleviated IS-induced neuroinflammation by modulating gut microbiota composition and inhibiting the TLR4/NF-κB signaling pathway. | (Sun et al., 2022) |
| *Gastrodia elata* Blume | ApoE−/− mice | unpredictable chronic mild stress | Serum corticosterone and amyloid-42 (Aβ-42) ↓; serotonin,,dopamine, and 3,4-dihydroxyphenylacetic acid in the prefrontal cortex ↑; fecal short-chain fatty acids (SCFAs), acetate, and butyrate ↓. | *Bifidobacterium, Akkermansia, Alloprevotella,* Defluviitaleaceae_UCG-011, and *Bifidobacterium, pseudolongum* ↑ | GE alleviated depression-like effects and cognitive impairment by improving intestinal microbiome and metabolite regulation. | (Huang et al., 2023) |
| *Eucommiae* *cortex* polysaccharides (EP) | ICR mice | obesogenic diet (OD)-induced cognitive and social dysfunction | QA and glutamate in the hippocampus ↓; microglial density and IL-1β, TNF-α, IL-6, NF-κB, TLR4, Inducible Nitric Oxide Synthase (iNOS), and LPS in the hippocampus ↓; Quinolinic Acid (QA) /Kynurenic Acid (KYNA) ratio ↓, while SCFAs and butyrate in the colon ↑; indole-3-acetic acid in the serum ↑. | F/B ratio ↑, *Clostridium butyricum, Fibrobacter, Roseburia, Butyrococcus, Fibrobacter*, and *Roseburia* ↑; *Escherichia coli* ↓ | EP remodeled gut microbiota composition, inhibited colitis and alleviated OD-induced neuroinflammation of abnormal neurocognitive and social behavior. | (Sun et al., 2022) |
| *Cistanche deserticola* polysaccharide ( CDPS ) | old male Kunming mice | Aging causes cognitive impairment | Glutathione Peroxidase (GSH-Px), Superoxide Dismutase (SOD), IL-10 (Interleukin-10) ↑, IL-2 (Interleukin-2), TNF-α, Advanced Oxidation Protein Products, and Lipid Peroxidation ↓. | Methanobacteria, Spirochaetia, Deltaproteobacteria,, Mollicutes, Nitrososphaeria, Anaerolineae, Erysipelotrichia↑;Thermoplasmata, Bacilli, Fusobacteriia↓ | CDPS improved the cognitive function of D-galactose-treated mice by restoring intestinal microbial homeostasis, thereby reducing oxidative stress and peripheral inflammation. | (Gao et al., 2021) |
| Saponins isolated from *Radix polygalae* (RPS) | C57BL/6 mice aged 30 weeks and Caenorhabditis elegans | Aging causes cognitive impairment | ROS in neural cells ↓; postsynaptic density protein 95 (PSD-95) and BDNF in the brain ↓. | Actinobacteria, Bacteroidetes, Firmicutes, *Robinsoniella, Dehalobacterium, Eubacterium, Lactobacillus,* and *Clostridium*↑; Proteobacteria, Verrucomicrobia, and *Akkermansia muciniphila*↓ | RPS modulated gut microbiota diversity, reduced neurological deficits in aging mouse models. | (Zeng et al., 2021) |
| Puerariae Lobatae Radix （PLR） with chuanxiong Rhizoma （CXR） | Male Sprague-Dawley rats | IS | Claudin-5 and ZO-1 levels in the brain ↑; Diamine Oxidase (DAO) , lipopolysaccharide, and D-lactic acid ↓. | *Alloprevotella,* Ruminococcaceae_UCG_005, NK4A214_group and UCG_004, *Oscillospira,* Lachnospiraceae_NK4B4_group, *Akkermansia, and Megasphaera*↑ | The combination of LR and CXR alleviated intestinal flora imbalance and brain-gut barrier damage, and treated cerebral IS. | (Chen et al., 2019) |
| Shouhui Tongbian Capsules (SHTB) | Rats | cerebral ischemic stroke | Nissl bodies ↑; ZO-1 and Occludin ↑; LPS and DAO in serum ↓. | Firmicutes, Lachnospiraceae*,* Lachnospiraceae, *Blautia, Coriobacteriia, Coriobacteriales, Eubacterium_oxidoreducens*_group*,* Christensenellaceae*, Christensenellales, Adlercreutzia,* Leuconostocaceae, *Weissella*, WPS-2, *Lactobacillus* ↑; Proteobacteria, *Escherichia-Shigella ↓.* | SHTB regulated the abundance of Escherichia coli and Lactobacillus, increased the expression of Peroxisome Proliferator-Activated Receptor Gamma (PPARγ) induced by SCFAs, improved lipid metabolism disorders, down-regulated lipid oxidative stress, inhibited ferroptosis, and reduced brain injury. | (Wei et al., 2025) |
| Huanglian Jiedu decoction （HLJDD) | Tg-APP/PS1 mice | AD | Amyloid-beta (Aβ) plaques in the cortex and hippocampus ↓; SOD, IL-4, IL-10, glutamate, GABA, arachidonic acid, DHA, eicosapentaenoic acid, linoleic acid, oleic acid, phosphatidylcholines (PCs), and phosphatidylethanolamines (PEs) in the brain ↑; IL-6, Malondialdehyde (MDA), L-cysteine, γ-linolenic acid, and 5-Lipoxygenase in the brain ↓; IL-1β, IL-6, and TNF-α in serum ↓; taurochenodeoxycholic acid, tauro-α-muricholic acid,, and deoxycholic acid ↓. | Firmicutes*,* Bacteroidales_*S24-7_group,* Lachnospiraceae and Porphyromonadaceae, Lachnospiraceae_NK4A136_group, *Bacteroides*, *Roseburia*, *Anaerotruncus* Lachnospiraceae_FCS020_group and *Odoribacter*↓; Proteobacteria*,* Prevotellaceae, Lactobacillaceae, Peptococcaceae, Alcaligenaceae, and Helicobacteraceae, Prevotellaceae_UCG_001, Lactic acid bacteria, *Helicobacter pylori,* Lachnospiraceae - UCG-001, Tyzzerella-3, Ruminococcaceae - UCG-014 and *Parasutterella*↑ | HLJDD inhibited gut microbiota dysbiosis and associated Aβ ，accumulation, alleviated neuroinflammation, and reversed cognitive impairment. | (Gu et al., 2021) |
| Luteolin | Sprague Dawley rat, Balb/c mice, Kunming mice | AD | In the cortex and hippocampus, Brain-Derived Neurotrophic Factor (BDNF), Tropomyosin Receptor Kinase B, synapsin I, and PSD-95 ↑, while reactive oxygen species (ROS) and lipid peroxidation ↓; in the cerebral cortex and hippocampus, NF-κB, β-secretase, astrocytes, and microglia ↓; in the brain, GSK-3β, p-Tau, TNF-α, and IL-1β ↓. | *Alistipes, Helicobacter,* and *Oscillibacter*↓ | Luteolin exerted therapeutic effects on AD by modulating brain glucose metabolism, regulating the gut microbiota-liver-brain axis, and attenuating neuroinflammation. | (Daily et al., 2021) |
| Resveratrol | C57BL/Cmice, ApoE-/-mice | Atherosclerosis (AS) | Serum Trimethylamine and Trimethylamine N-Oxide (TMAO) ↓, BAs in the liver, flavin-containing monooxygenase ↑; TMAO, primary BAs, taurocholic acid, tauro-β-muricholic acid, cholic acid, chenodeoxycholic acid, and deoxycholic acid (DCA) ↓. | Firmicutes*, Lactobacillus, Bifidobacterium* and *Akkermansia*↑；*Prevotella,* Ruminococcaceae_unclas  sified *and Bilophila*↓ | Resveratrol attenuated TMAO-induced AS by reshaping the gut microbiota to reduce TMAO levels and enhancing BA neosynthesis partially mediated by the gut-liver FXR-FGF15 axis. | (Chen et al., 2016) |
| Qifu Yin | KM mice | memory impairment (MI) | In brain tissue, SOD, and GSH-Px ↑, while MDA ↓; in the hippocampus, CREB and BDNF ↑, whereas recombinant amyloid precursor protein (APP), apoptosis signal-regulating kinase-1 (ASK1), and c-Jun N-terminal kinase (JNK) mRNA ↓; in serum, N-methyl-D-aspartate receptor (NMDAR) ↓ and CREB ↑. | Fusobacteria, Planctomycetes, and Verrucomicrobia, *Akkermansia, Paramuribaculum, Herminiimonas, Erysipelatoclostridium*↑ | Qifu Yin improved memory in HBM-induced MI animals by restoring intestinal flora homeostasis and regulating relevant indicators in serum and brain tissue. | (Liu et al., 2024) |

References:

Chen, M., Yi, L., Zhang, Y., Zhou, X., Ran, L., Yang, J., et al. (2016). Resveratrol Attenuates Trimethylamine-N-Oxide (TMAO)-Induced Atherosclerosis by Regulating TMAO Synthesis and Bile Acid Metabolism via Remodeling of the Gut Microbiota. *Mbio* 7, e02210-2215. doi: 10.1128/mBio.02210-15

Chen, R., Wu, P., Cai, Z., Fang, Y., Zhou, H., Lasanajak, Y., et al. (2019). Puerariae Lobatae Radix with chuanxiong Rhizoma for treatment of cerebral ischemic stroke by remodeling gut microbiota to regulate the brain-gut barriers. *J Nutr Biochem* 65, 101–114. doi: 10.1016/j.jnutbio.2018.12.004

Daily, J. W., Kang, S., and Park, S. (2021). Protection against Alzheimer’s disease by luteolin: Role of brain glucose regulation, anti-inflammatory activity, and the gut microbiota-liver-brain axis. *Biofactors* 47, 218–231. doi: 10.1002/biof.1703

Fan, X., Dong, W., Huang, Y., Shu, Y., Yan, Y., Mi, J., et al. (2024). Aqueous extract of lycium ruthenicum murray attenuates neuroinflammation in C57BL/6J mice induced by high-fat and high-fructose diet through regulating gut microbiota and bile acid metabolism. *Foods (basel Switz.)* 13, 3812. doi: 10.3390/foods13233812

Fu, J., Li, J., Sun, Y., Liu, S., Song, F., and Liu, Z. (2023). In-depth investigation of the mechanisms of Schisandra chinensis polysaccharide mitigating Alzheimer’s disease rat via gut microbiota and feces metabolomics. *Int J Biol Macromol* 232, 123488. doi: 10.1016/j.ijbiomac.2023.123488

Gao, Y., Li, B., Liu, H., Tian, Y., Gu, C., Du, X., et al. (2021). Cistanche deserticola polysaccharides alleviate cognitive decline in aging model mice by restoring the gut microbiota-brain axis. *Aging (Albany NY)* 13, 15320–15335. doi: 10.18632/aging.203090

Gu, X., Zhou, J., Zhou, Y., Wang, H., Si, N., Ren, W., et al. (2021). Huanglian Jiedu decoction remodels the periphery microenvironment to inhibit Alzheimer’s disease progression based on the “brain-gut” axis through multiple integrated omics. *Alzheimers Res Ther* 13, 44. doi: 10.1186/s13195-021-00779-7

Hu, K., Zhou, Z., Li, H., Xiao, J., Shen, Y., Ding, K., et al. (2025). Regulation of histidine metabolism by Lactobacillus Reuteri mediates the pathogenesis and treatment of ischemic stroke. *Acta Pharm Sin B* 15, 239–255. doi: 10.1016/j.apsb.2024.10.003

Huang, H.-S., Lin, Y.-E., Panyod, S., Chen, R.-A., Lin, Y.-C., Chai, L. M. X., et al. (2023). Anti-depressive-like and cognitive impairment alleviation effects of Gastrodia elata Blume water extract is related to gut microbiome remodeling in ApoE-/- mice exposed to unpredictable chronic mild stress. *J Ethnopharmacol* 302, 115872. doi: 10.1016/j.jep.2022.115872

Liu, S., Zhang, Q., Zhao, F., Deng, F., and Wang, Y. (2024). Regulating effect of Qifu Yin on intestinal microbiota in mice with memory impairment induced by scopolamine hydrobromide. *J Ethnopharmacol* 333, 118445. doi: 10.1016/j.jep.2024.118445

Song, J., Li, M., Kang, N., Jin, W., Xiao, Y., Li, Z., et al. (2024). Baicalein ameliorates cognitive impairment of vascular dementia rats via suppressing neuroinflammation and regulating intestinal microbiota. *Brain Res Bull* 208, 110888. doi: 10.1016/j.brainresbull.2024.110888

Sun, P., Wang, M., Li, Z., Wei, J., Liu, F., Zheng, W., et al. (2022). Eucommiae cortex polysaccharides mitigate obesogenic diet-induced cognitive and social dysfunction via modulation of gut microbiota and tryptophan metabolism. *Theranostics* 12, 3637–3655. doi: 10.7150/thno.72756

Wei, F., Zhou, J., Pan, L., Shen, M., Niu, D., Zeng, Z., et al. (2025). Integrative microbiomics, proteomics and lipidomics studies unraveled the preventive mechanism of Shouhui Tongbian Capsules on cerebral ischemic stroke injury. *J Ethnopharmacol* 337, 118874. doi: 10.1016/j.jep.2024.118874

Zeng, W., Wu, A. G., Zhou, X.-G., Khan, I., Zhang, R. L., Lo, H. H., et al. (2021). Saponins isolated from Radix polygalae extent lifespan by modulating complement C3 and gut microbiota. *Pharmacol Res* 170, 105697. doi: 10.1016/j.phrs.2021.105697

Zhang, C., Zhang, Y., Zhao, T., Mou, T., Jing, W., Chen, J., et al. (2022). Schisandrin alleviates the cognitive impairment in rats with Alzheimer’s disease by altering the gut microbiota composition to modulate the levels of endogenous metabolites in the plasma, brain, and feces. *Front Pharmacol* 13, 888726. doi: 10.3389/fphar.2022.888726
