# Supplementary material for: Synergistic therapy of Chinese herbal medicine and gut microbiota modulation for post-stroke cognitive recovery: focus on microbial metabolite and immunoinflammation
Source: Front Microbiol. 2025 Aug 14;16:1623843. doi: 10.3389/fmicb.2025.1623843 (PMC12391124; doi:10.3389/fmicb.2025.1623843)
Supplement: Supplementary file 2 [file Table_2.docx]

Supplementary Material

**Table S2. Microbiota-targeted interventions for post-stroke cognitive impairment and associated comorbidities.**

| Intervention | | Species/Strain | Disease | Effects on related biomarker | Intestinal microbiota modulation | Observations/ Significance | Reference |
| --- | --- | --- | --- | --- | --- | --- | --- |
| Probiotics | *Clostridium butyricum* (*C. butyricum*) | Male C57BL/6 mice | Diabetes-induced cerebral ischemia/reperfusion injury | In brain tissue, phosphorylated protein kinase B (p-Akt) **↑** and caspase-3**↓**. | Fecal microbiota diversity**↑**, *Clostridiumcluster* XIVab*, F. prausnitzii, Bifidobacterium, and Lactobacillus* **↑**; *Clostridium cluster* XI*, Clostridium cluster* I*,* Enterobacteriaceae and *Enterococcus* spp **↓** | *C. butyricum* alleviates microglia-mediated neuroinflammation by modulating the gut microbiota-gut-brain axis mediated by the metabolite butyrate. | (Sun et al., 2016) |
|  | *Lactobacillus* *reuteri* GMNL-89, *Lactobacillus paracasei* GMNL-133 | C57BL/6 mice | Ischemic Stroke (IS) | Evans blue dye extravasation in the right hemisphere **↓**; claudin-5 and cyclooxygenase-2 (COX-2) in the brain **↓**. claudin-2 and claudin-3 in the intestine **↑**, while interleukin-6 (IL-6) and tumor necrosis factor alpha (TNF-α) in the intestine **↓**; plasma endotoxin,interleukin-1 beta (IL-1β), IL-6, and TNF-α**↓**, whereas neutrophil **↑**; total short-chain fatty acids (SCFAs), acetate, butyrate, and propionate **↑**. | Firmicutes / Bacteroidete (F/B) **↑**, *Clostridia, Clostridiales,* *L. reuteri and L. paracasei* **↑**; *Coprococcus, Lachnospira*, *Lactonifactor, Lachnospira pectinoschiza , Lactonifactor longoviformis* **↑** | G89 and G133 exert protective effects against ischemic stroke by modulating the gut-spleen-brain axis | (Wang et al., 2025) |
|  | *Lactobacillus* and *Bifidobacterium*, *Lactobacillus rhamnosus* GG（LGG） | Male C57 BL/6 mice | atherosclerosis | Trimethylamine (TMA) in the cecum and serum **↓**, while serum trimethylamine N-Oxide (TMAO), triglycerides (TG), and farnesoid X receptor (FXR)**↓**; cholesterol 7 alpha-hydroxylase **↑** | The bacterial strain F1-3-2 colonizes the gut. | The strain was participate in the TMA–TMAO regulation, improve the lipid metabolism, and alleviate atherosclerosis caused by TMAO | (Liang et al., 2020) |
|  | *C. butyricum* | APP/PS1 transgenic mice | AD | Aβ deposition, microglia, TNF-α and IL-1β, Cluster of Differentiation 11b and COX-2 **↓**; p-Phosphorylated Nuclear Factor Kappa B (NF-κB) in BV2 microglial cells **↓**. | Deferribacteres *Alloprevotella and* S24-7 **↑**; Helicobacteraceae*, Helicobacter* **↓** | CB treatment attenuated microglia-mediated neuroinflammation via regulating the GM–gut–brain axis, which was mediated by the metabolite butyrate. | (Sun et al., 2020) |
|  | *Akkermansia muciniphila* | C57BL/6J mice | sleep-deprived cognitive impairment | Synaptic proteins vesicular glutamate transporter 1 (VGLUT1) and postsynaptic density protein 95 (PSD-95), acetate and butyrate **↑** in the hippocampus; synaptophysin (SYP), VGLUT1, and PSD-95 in the dentate gyrus**↑**;, SYP puncta **↓** | A. muciniphila colonization **↑** | Mucinophilic fusobacteria attenuate microglia-mediated neuroinflammation by modulating the mediated gut-brain axis mediated by the metabolites butyrate and butyric acid. | (Li et al., 2023) |
| Faecal microbiota transplantation | Feces from young mice | C57BL/6 mice | IS | Claudin-1 and occludin **↑** in the colon, vascular endothelial growth factor (VEGF), prospero homeobox protein 1 (PROX1) in the cerebral cortex **↑**; acetate levels in feces, serum, and the cerebral cortex **↑**. | *Bacteroides* **↓** | Transplantation of young microbiota ameliorates stroke by enhancing angiogenesis and lymphatic inward growth. | (Yuan et al., 2024) |
|  | feces from cognitively normal rats | SD rat pups | Neonatal hypoxic-ischemic brain damage (HIBD) | Interleukin-17A (IL-17A) and interleukin-22 (IL-22) in the colon **↑**, while occludin and  zonula occludens-1 (ZO-1) **↓**; Ionized calcium-binding adapter molecule 1 (IBA-1) and Glial fibrillary acidic protein (GFAP-positive cells) in the Carbonic anhydrase 1 (CA1) region of the hippocampus, as well as dendritic spine and branching density**↑**. | *Akkermansiaceae, Enterobacteriaceae***↑** | Transplantation of a healthy gut microbiota enables early correction of gut microbial dysbiosis and gut dysfunction, and mitigates long-term cognitive deficits caused by neonatal HIBD. | (Chen et al., 2025) |
|  | Feces of mice carried after antibiotic treatment | C57BL/6 | IS | Regulatory T cells **↑**, IL-17-positive γδ T cells **↓**. CD4⁺ FoxP3⁺ Tregs in the lamina propria of the small intestine **↑**, IL-17⁺ γδ T cells **↓**, CD45 high infiltration **↓**. | Microbial diversity **↓**; Clostridiales and Bacteroidetes S24-7 **↓**; *Lachnospiraceae, Verrucomicrobiaceae and Anaeroplasmataceae* **↑** | Intestinal bacterial alterations lead to localized T reg amplification and effector IL-17 + γδ T cell suppression in the small intestine, and intestinal T cells are translocated to the meninges for neuroprotection. | (Benakis et al., 2016) |
|  | Sham-operated rat feces | SD rats | bilateral common carotid artery occlusion. | The acetate content in hippocampal tissue, as well as SYP, PSD-95, n-methyl-d-aspartate receptor subunit 1, and syntaxin-1A **↑**, while histone deacetylase 6 **↓**; claudin-5 and occludin **↑**, whereas Reactive Oxygen Species (ROS) **↓**, Acetyl coenzyme A content in the hippocampus **↑**, and acetate and propionate in feces **↑**. | *Lactobacillus johnsonii, Akkermansia muciniphila, Ruminococcus, Clostridia*_UCG_014 **↑**. | The gut microbiota of healthy mice exerts neuroprotective effects against chronic cerebral hypoperfusion by inhibiting HDAC. | (Su et al., 2023) |
|  | Healthy human feces | Human | Aging-related cognitive impairment | The montreal cognitive assessment-B (MoCA-B), activities of daily living (ADL) **↓**; bilirubin, 4-hydroxypheoxyacetater, phloracetophenone, α-furfuryl diketone, and squamaldehyde A **↓**. 3β,12α-dihydroxy-5α-cholanic acid, deoxycholic acid, and p-anisic acid **↑**. | s_un_g_Lachnospira and g_*Lachnospira* **↓**; c_Fusobacteriia, g_Prevotella_7, s_un_g_Provotella_7, and f_Eggerthellaceae**↑** | FEMT improves gut microbiota composition, influences differential metabolites and metabolic pathways, and alleviates cognitive impairment. | (Chen et al., 2023) |
| Prebiotics | Xylo-oligosaccharides (XOS) | APP/PS1 mice | Cognitive dysfunction | IL-1β, IL-6, and interleukin-10 (IL-10) in the colon and hippocampus **↓**; ZO-1, occludin in the hippocampus **↑**. IBA-1 positive cells **↓**. | Melainabacteria, order Gastranaerophilales, family Prevotellaceae, genus *Alloprevotella*, A2, and Ruminococcaceae_UCG_009, *Muribaculum* and *Lactobacillus* **↑**, while Eggerthellaceae **↓**. | XOS intervention effectively alleviated surgery-induced cognitive impairment and gut microbiota alterations, reduced inflammatory responses, and improved the integrity of tight junction barriers in the gut and hippocampus. | (Han et al., 2020) |
|  | Fructooligosaccharides (FOS) | APP/PS1 transgenic (Tg) mice, SD rats, and C57 mice | AD | Synaptophysin I and PSD-95 in the brain **↑**, while phosphorylated c-Jun N-terminal Kinase and amyloid-beta 42 (Aβ42) **↓**. lucagon-Like peptide-1 (GLP-1) in the gut **↑**; SOD **↑**, Na⁺/K⁺-ATPase activity **↑**, and acetylcholine (ACh) **↑;** interferon gamma (IFN-γ); IL-10, IL-12, IL-17A, IL-4, TNF-α, and VEGF **↓**; norepinephrine (NE), dopamine (DA), Serotonin (5-HT), and 5-hydroxyindoleacetic acid (5-HIAA) **↑**. | Clostridium and Clostridiaceae, Proteobacteria, Epsilonproteobacteria, Helicobacteraceae, Deferribacteraceae, and Helicobacter **↓**; Firmicutes, Bacteroidetes, *Bacteroides, Bacillus, Lactobacillus,* Lactobacillaceae **↑**. | FOS exerts beneficial effects on AD by modulating the gut microbiota–GLP-1/GLP-1R pathway. | (Chen et al., 2017; Sun et al., 2019) |
|  | Chitooligosaccharides (COS) | Male C57BL/6J mice | Hepatic encephalopathy (HE) | GSH and T-SOD levels in brain tissue **↑**. In the hippocampus, IBA-1, IL-1β **↑**, while microglia and astrocytes **↓**; In the cerebral cortex, IL-1β, IL-6, monocyte chemoattractant protein-1 (MCP-1), and TNF-α **↓**; along with caspase-3, caspase-9, TLR4, myeloid differentiation factor (MyD88), and p-NF-κB **↓**. | Muribaculaceae, *Lactobacillus* and *Enterorhabdus,* Actinobacteriota**↓**; | COS inhibits neuroinflammation and hepatitis by downregulating the Toll-like receptor 4/nuclear factor-kappa B (TLR4/NF-κB) pathway. | (Liu et al., 2023) |
|  | Yeast β-glucan | Mouse | AD | Hippocampal neurons **↑**, while p-tau and Aβ **↓**. | *Lactobacillus, Bifidobacterium*, Saccharibacteria_genera_incertae_sedis, and Desulfovibrionaceae **↑**; *Oscillibacter, Mucispirillum, Alistipes*, *Anaerotruncus, Rikenella*, and *Butyricicoccus* **↓** | Yeast β-glucan modulates gut microbiota composition, alleviates neuroinflammation, and enhances cognitive function. | (Xu et al. 2020) |
|  | Oligosaccharides | Swiss albino male mice | Cognitive decline mediated by intestinal malnutrition | Hippocampal acetylcholinesterase (AChE) **↓**; myeloperoxidase in the colon and brain **↓**; hippocampal SOD **↑**, TNF-α, IL-1β, and C-C motif chemokine ligand 2 (CCL2) **↓**; hippocampal neurons **↑.**. | Lactobacillus, Bifidobacterium, Firmicutes, and *Clostridium***↑** | Xylo-oligosaccharides (XOS) and quercetin effectively alleviate antibiotic-associated gut microbiota dysbiosis and prevent dysbiosis-related cognitive impairment in mice. | (Sarkar et al., 2022) |
|  | Fructooligosaccharides (FOS) and Galactooligosaccharides (GOS) | 2-month-old male C57BL/6J mice | High-fat diet-induced age-related cognitive decline | Microglial phagocytosis and C-C Chemokine receptor type 2 in the brain **↑**; microglial ROS,TNF-α **↓**; CCL2, IL-10 **↑** | Muribaculaceae, Prevotellaceae, Rikenellaceae and Oscillospiraceae **↑** | Prebiotics regulate Triggering Receptor Expressed on Myeloid cells 2, delay microglial senescence, exert anti-inflammatory effects, and alleviate cognitive impairment. | (Vijaya et al., 2024) |
| Postbiotics | Bifidobacterium lactis subsp. IOBL07, Lactiplantibacillus plantarum IOB602, and Lactobacillus paracasei IOB413 ( inactivated) | Wistar rats | AD | Aβ in the hippocampus **↓**; LPS in feces, serum, and hippocampus **↓**; TLR4, NLR Family Pyrin Domain Containing 3, and MyD88 in the brain **↓**. | Firmicutes and Ruminococcaceae **↑**; Muribaculaceae **↓** | Postbiotics improve the structural composition of the gut microbiota, maintain the integrity of the small intestinal mucosal barrier, and downregulate inflammation in brain tissue, exerting neuroprotective effects. | (Xiao et al., 2025) |
|  | *Lactobacillus plantarum* -derived postbiotics | Male C57BL/6 mice | Salmonella-induced neurological dysfunction | Inflammatory cell in the brain **↓**, IL-1β, IL-6, and TNF-α**↓**, IL-4 and IL-10**↑**; 5-HT, Brain-Derived Neurotrophic Factor, DA, neuropeptide Y, and acetylcholine in the brain and serum **↑**; acetate and propionate **↑**. | *Helicobacter pylori*, *Lactobacillus*, and *Dubosiella* **↑**; *Mucispirillum*, norank_f_Oscillospiraceae, and Eubacterium_siraeum_group **↓** | LP postbiotics optimize gut microbiota composition and SCFAs to suppress neuroinflammation, ultimately contributing to the alleviation of Salmonella-induced neurological dysfunction. | (Wu et al., 2022) |
|  | cell-free supernatant (CFS) obtained from probiotics | SD rats | IS | Pyknotic or dark neurons in cortex **↓**; glial fibrillary acidic protein, TLR-4, TNF-α, IL-1β, IL-6 and matrix metallo proteinase 9 (MMP9) in brain **↓**; NeuN ZO-1 in brain **↑**; occludin, ZO-1 in cortex **↑**; TNF-α, IL-1β, and MMP9 in Blood **↓** | Proteobacteria and Actinobacteria **↓**; F/B **↑** | The ingestion of probiotic-derived CFS may provide neuroprotective effects by inhibiting neuroinflammation and modulating gut-brain communication function. | (Rahman et al., 2024) |

References:

Benakis, C., Brea, D., Caballero, S., Faraco, G., Moore, J., Murphy, M., et al. (2016). Commensal microbiota affects ischemic stroke outcome by regulating intestinal γδ T cells. *Nat Med* 22, 516–523. doi: 10.1038/nm.4068

Chen, A., Teng, C., Wei, J., Wu, X., Zhang, H., Chen, P., et al. (2025). Gut microbial dysbiosis exacerbates long-term cognitive impairments by promoting intestinal dysfunction and neuroinflammation following neonatal hypoxia-ischemia. *Gut Microbes* 17, 2471015. doi: 10.1080/19490976.2025.2471015

Chen, D., Yang, X., Yang, J., Lai, G., Yong, T., Tang, X., et al. (2017). Prebiotic Effect of Fructooligosaccharides from Morinda officinalis on Alzheimer’s Disease in Rodent Models by Targeting the Microbiota-Gut-Brain Axis. *Front Aging Neurosci* 9, 403. doi: 10.3389/fnagi.2017.00403

Chen, X., Zhang, W., Lin, Z., Zheng, C., Chen, S., Zhou, H., et al. (2023). Preliminary evidence for developing safe and efficient fecal microbiota transplantation as potential treatment for aged related cognitive impairments. *Front Cell Infect Microbiol* 13, 1103189. doi: 10.3389/fcimb.2023.1103189

Han, D., Li, Z., Liu, T., Yang, N., Li, Y., He, J., et al. (2020). Prebiotics Regulation of Intestinal Microbiota Attenuates Cognitive Dysfunction Induced by Surgery Stimulation in APP/PS1 Mice. *Aging Dis* 11, 1029–1045. doi: 10.14336/AD.2020.0106

Li, N., Tan, S., Wang, Y., Deng, J., Wang, N., Zhu, S., et al. (2023). Akkermansia muciniphila supplementation prevents cognitive impairment in sleep-deprived mice by modulating microglial engulfment of synapses. *Gut Microbes* 15, 2252764. doi: 10.1080/19490976.2023.2252764

Liang, X., Zhang, Z., Lv, Y., Tong, L., Liu, T., Yi, H., et al. (2020). Reduction of intestinal trimethylamine by probiotics ameliorated lipid metabolic disorders associated with atherosclerosis. *Nutrition* 79–80, 110941. doi: 10.1016/j.nut.2020.110941

Liu, P., Li, H., Xu, H., Gong, J., Jiang, M., Qian, J., et al. (2023). Chitooligosaccharides Attenuated Hepatic Encephalopathy in Mice through Stabilizing Gut-Liver-Brain Disturbance. *Mol Nutr Food Res* 67, e2200158. doi: 10.1002/mnfr.202200158

Rahman, Z., Padhy, H. P., and Dandekar, M. P. (2024). Cell-free supernatant of lactobacillus rhamnosus and bifidobacterium breve ameliorates ischemic stroke-generated neurological deficits in rats. *Probiotics Antimicrob. Proteins*. doi: 10.1007/s12602-024-10256-w

Sarkar, S. R., Mazumder, P. M., and Banerjee, S. (2022). Oligosaccharide and Flavanoid Mediated Prebiotic Interventions to Treat Gut Dysbiosis Associated Cognitive Decline. *J Neuroimmune Pharmacol* 17, 94–110. doi: 10.1007/s11481-021-10041-4

Su, S.-H., Chen, M., Wu, Y.-F., Lin, Q., Wang, D.-P., Sun, J., et al. (2023). Fecal microbiota transplantation and short-chain fatty acids protected against cognitive dysfunction in a rat model of chronic cerebral hypoperfusion. *CNS Neurosci Ther* 29 Suppl 1, 98–114. doi: 10.1111/cns.14089

Sun, J., Liu, S., Ling, Z., Wang, F., Ling, Y., Gong, T., et al. (2019). Fructooligosaccharides Ameliorating Cognitive Deficits and Neurodegeneration in APP/PS1 Transgenic Mice through Modulating Gut Microbiota. *J Agric Food Chem* 67, 3006–3017. doi: 10.1021/acs.jafc.8b07313

Sun, J., Wang, F., Ling, Z., Yu, X., Chen, W., Li, H., et al. (2016). Clostridium butyricum attenuates cerebral ischemia/reperfusion injury in diabetic mice via modulation of gut microbiota. *Brain Res* 1642, 180–188. doi: 10.1016/j.brainres.2016.03.042

Sun, J., Xu, J., Yang, B., Chen, K., Kong, Y., Fang, N., et al. (2020). Effect of Clostridium butyricum against Microglia-Mediated Neuroinflammation in Alzheimer’s Disease via Regulating Gut Microbiota and Metabolites Butyrate. *Mol Nutr Food Res* 64, e1900636. doi: 10.1002/mnfr.201900636

Vijaya, A. K., Kuras, S., Šimoliūnas, E., Mingaila, J., Makovskytė, K., Buišas, R., et al. (2024). Prebiotics Mitigate the Detrimental Effects of High-Fat Diet on memory, anxiety and microglia functionality in Ageing Mice. *Brain Behav Immun* 122, 167–184. doi: 10.1016/j.bbi.2024.08.022

Wang, Y.-H., Liao, J.-M., Jan, M.-S., Wang, M., Su, H.-H., Tsai, W.-H., et al. (2025). Prophylactic use of probiotics as an adjunctive treatment for ischemic stroke via the gut-spleen-brain axis. *Brain Behav Immun* 123, 784–798. doi: 10.1016/j.bbi.2024.10.026

Wu, Y., Wang, Y., Hu, A., Shu, X., Huang, W., Liu, J., et al. (2022). Lactobacillus plantarum-derived postbiotics prevent Salmonella-induced neurological dysfunctions by modulating gut-brain axis in mice. *Front Nutr* 9, 946096. doi: 10.3389/fnut.2022.946096

Xiao, L., Tang, L., Song, X., Zhang, Y., Han, X., Lv, H., et al. (2025). Postbiotics regulate intestinal microbiota and reduce Aβ deposition in the brain to improve cognitive impairment in AD rats. *Food Science* 46, 182–193.

Yuan, Y., Li, L., Wang, J., Myagmar, B.-O., Gao, Y., Wang, H., et al. (2024). Gut microbiota-derived acetate promotes long-term recovery through angiogenesis guided by lymphatic ingrowth in older adults with stroke. *Front Neurosci* 18, 1398913. doi: 10.3389/fnins.2024.1398913
