# Supplementary material for: Synergistic therapy of Chinese herbal medicine and gut microbiota modulation for post-stroke cognitive recovery: focus on microbial metabolite and immunoinflammation
Source: Front Microbiol. 2025 Aug 14;16:1623843. doi: 10.3389/fmicb.2025.1623843 (PMC12391124; doi:10.3389/fmicb.2025.1623843)
Supplement: Supplementary file 3 [file Table_3.docx]

Supplementary Material

**Table S3. Effects of combination therapy on post-stroke cognitive impairment and its associated comorbidities.**

| Drugs | Species/Strain | Disease | Effects on related biomarkers | Intestinal microbiota modulations | Observations/ Significance | References |
| --- | --- | --- | --- | --- | --- | --- |
| Eleutheroside E and Eleutheroside E donor fecal transplantation. | KM mice | Radiation-induced cognitive impairment. | Serotonin (5-HT) and acetylcholine (ACh), Norepinephrine (NE) in serum, as well as Brain-Derived Neurotrophic Factor (BDNF) and Phosphorylated cAMP Response Element-Binding Protein (p-CREB) in the colon and hippocampus **↓**; while Gamma-Aminobutyric Acid (GABA) **↑**; Occludin, Zonula Occludens-1 (ZO-1), and Claudin in the colon **↑**. | *Phascolarctobacterium* and *Lactobacillus* **↑**, while *Helicobacter, Alloprevotella, Treponema*, *Streptococcus*, *Odoribacter*, *Ruminococcus*, *Aquabacterium*, and Proteobacteria **↓**. | EE alleviates radiation-induced cognitive and memory impairment by activating the PKA/CREB/BDNF signaling pathway through gut microbiota. | (Song et al., 2022) |
| Huayu Ditan Yizhi Formula combined with cognitive rehabilitation training. | human | Post-stroke cognitive impairment (PSCI). | 5-HT, dopamine (DA), and NE in serum **↓**. | OUT, Chao1, and Shannon index **↑**, while Simpson index **↓**. | The formula combined with cognitive rehabilitation training improves gut microbiota diversity, increases neurotransmitter levels, and significantly enhances cognitive function in PSCI patients. | (Wang et al., 2024) |
| Xylo-oligosaccharides (XOS), Lactobacillus paracasei HII01. | Male Wistar rats. | Cognitive impairment associated with obesity-induced insulin resistance. | Serum Lipopolysaccharide (LPS) **↓**, dendritic spine density **↑**, hippocampal Bax **↑**; and B-cell lymphoma 2 (Bcl-2) **↓**; microglial normalization **↑**; colonic interleukin-1 beta (IL-1β) and interleukin-6 (IL-6) **↓**. | Firmicutes/Bacteroidetes (F/B ) ratio**↓** | The consumption of prebiotics, probiotics, and synbiotics restored cognitive function in obese insulin-resistant subjects through the gut-brain axis. | (Chunchai et al., 2018) |
| Feces from healthy adults, Bifidobacterium quadruple viable tablets. | Human | Alzheimer's disease (AD) | AD assessment scale-cognitive subscale score, activities of daily living scale score **↓**; mini-mental state examination score **↑**; serum TNF-α, IL-6, Malondialdehyde (MDA), Diamine Oxidase, homocysteine, endotoxin, and urinary lactulose/mannitol ratio **↓**; Superoxide Dismutase (SOD) **↑**. |  | Fecal microbiota transplantation combined with probiotic therapy significantly improved cognitive function and gut barrier function in elderly patients with moderate AD. | (Fan et al., 2025) |
| Lactobacillus reuteri (L. reuteri), Cornus officinalis | C57BL/6 mice | Cognitive impairment | Brain tissue MDA, ACh, acetylcholinesterase, phosphorylated tau protein, Amyloid-beta, and caspase-7 **↓**; SOD and Choline Acetyltransferase (ChAT) **↑**.  Serum albumin **↑**, ITC-dextran content **↓**; Colon tissue occludin and claudin-1 **↑**; MPO, TLR4, phosphorylated IκBα (p-IκBα), phosphorylated nuclear factor kappa B (p-NF-κB), TNF-α, caspase-1, IL-1β, Inducible Nitric Oxide Synthase, and Cyclooxygenase-2 (COX-2) **↓**; Fecal acetate, propionate, and butyrate **↑**., | Lactobacillaceae, *Lactobacillus,* *Akkermensia muciniphila*, and *Alistipes onderdonkii* **↑**, while Proteobacteria, Firmicutes/Bacteroidetes ratio, and Prevotellaceae UCG-001 **↓**. | Synbiotic supplementation alleviated colitis and cognitive impairment by modulating the gut microbiota, pro-inflammatory cytokines, and  Short-Chain Fatty Acids. | (Lee et al., 2022) |

References:

Chunchai, T., Thunapong, W., Yasom, S., Wanchai, K., Eaimworawuthikul, S., Metzler, G., et al. (2018). Decreased microglial activation through gut-brain axis by prebiotics, probiotics, or synbiotics effectively restored cognitive function in obese-insulin resistant rats. *J Neuroinflammation* 15, 11. doi: 10.1186/s12974-018-1055-2

Fan, S., Wang, T., and Wen, G. (2025). Clinical efficacy of fecal microbiota transplantation combined with probiotics for moderate elderly AD patients. *Chinese Journal of Geriatric Heart Brain and Vessel Diseases* 27, 192–196.

Lee, H. L., Kim, J. M., Moon, J. H., Kim, M. J., Jeong, H. R., Go, M. J., et al. (2022). Anti-Amnesic Effect of Synbiotic Supplementation Containing Corni fructus and Limosilactobacillus reuteri in DSS-Induced Colitis Mice. *Int J Mol Sci* 24, 90. doi: 10.3390/ijms24010090

Song, C., Duan, F., Ju, T., Qin, Y., Zeng, D., Shan, S., et al. (2022). Eleutheroside E supplementation prevents radiation-induced cognitive impairment and activates PKA signaling via gut microbiota. *Commun Biol* 5, 680. doi: 10.1038/s42003-022-03602-7

Wang J., Cheng W., and Cheng G. (2024). Clinical efficacy of huayu ditan yizhi formula combined with cognitive rehabilitationtraining on post-stroke cognitive impairment and its effect on gut microbiota diversity. *Information on Traditional Chinese Medicine* 41, 47-51+57.
